# Bladder cancer subtypes exhibit limited plasticity across different microenvironments and in metastases

**DOI:** 10.1186/s40164-025-00682-z

**Published:** 2025-07-02

**Authors:** Carina Bernardo, Subhayan Chattopadhyay, Natalie Andersson, Pontus Eriksson, Benjamin Medle, Lena Tran, Nour al dain Marzouka, Adam Mattsson, Aymeric Zadoroznyj, Malin Larsson, Fredrik Liedberg, Mattias Höglund, Gottfrid Sjödahl

**Affiliations:** 1 Division of Oncology, Department of Clinical Sciences Lund, Lund University, Scheelevägen 2, Medicon Village, Lund, SE-223 81 Sweden; 2 Division of Clinical Genetics, Department of Laboratory Medicine, Lund University, Lund, Sweden; 3 Department of Pathology, Office of Medical Services, Region Skåne, Lund, Sweden; 4 Center for Biotechnology & Department of Biomedical Engineering, Khalifa University of Science and Technology, Abu Dhabi, United Arab Emirates; 5 Department of Physics, Chemistry and Biology, Science for Life Laboratory, National Bioinformatics Infrastructure Sweden, Linköping University, Linköping, Sweden; 6 Division of Urological Research, Department of Translational Medicine, Lund University, Skåne University Hospital, Malmö, Sweden

**Keywords:** Bladder cancer, Plasticity, Molecular subtypes, Tumor microenvironment, Metastasis, Tumor evolution, Genomics, Transcriptomics, Patient-derived xenografts

## Abstract

**Background:**

Transcriptomic and genomic analyses of bladder cancer (BC) reveal a highly diverse disease stratified into molecular subtypes with distinct molecular features and biological behaviors. Intratumor heterogeneity (ITH) and plasticity can significantly impact diagnosis and patient management, yet their extent in BC remains highly debated. Here, we investigated whether the three main bladder cancer subtypes maintain or alter their identity in response to changes in the microenvironment and during metastatic colonization.

**Methods:**

Seven patient-derived xenograft (PDX) models representing the major BC subtypes were propagated into three distinct tissue microenvironments: subcutaneous, mammary fat pad and under the kidney capsule. Metastatic lesions were generated via systemic injection of tumor cells. Tumor samples were analysed using RNA- and exome sequencing, SNP-arrays and histopathology to assess subtype fidelity, genomic evolution, and clonal dynamics.

**Results:**

A comprehensive, longitudinal multiomics analysis showed that tumors consistently maintain their molecular subtype, as well as their transcriptomic and genomic profiles, across different environments. No evidence of emerging ITH or subtype transitions was observed, regardless of the microenvironment. The transcriptomic adaptations observed in metastases and different implantation sites are limited and are associated primarily with hypoxia, epithelial-mesenchymal transition (EMT), and invasion.

**Conclusions:**

Our results suggest that invasive bladder cancers have a strong intrinsic tumor identity that is not easily reprogrammed by the microenvironment.

**Supplementary Information:**

The online version contains supplementary material available at 10.1186/s40164-025-00682-z.

## Introduction

Bladder cancer (BC) is the ninth most common cancer globally, with an estimated 614,000 new cases and 220,000 deaths in 2022 [1]. Most patients are diagnosed with non-muscle invasive tumors (NMIBCs), which have a good prognosis but a high recurrence rate. In contrast, muscle invasive BC (MIBC) has a high risk of metastasis and poor outcomes, with fewer than 50% of patients surviving 5 years despite curative-intent treatment with radical cystectomy, with or without perioperative systemic chemo- or immunotherapy [2, 3]. Thus, there is a need for better clinical stratification of MIBC patients to tailor more aggressive treatment regimens to those most likely to benefit.

Recent genomic and transcriptomic profiling of large tumor cohorts has highlighted the heterogeneity of BC, leading to the classification of MIBC into molecular subtypes [4]. Several classification systems are driven by varying degrees of immune/stromal content in the analyzed tissue samples [5–7]. In contrast, the Lund Taxonomy (LundTax) classification defines five intrinsic subtypes on the basis of the phenotypic states of the cancer cells: Urothelial-like (Uro), Genomically Unstable (GU), Basal/Squamous-like (BaSq), Small cell Neuroendocrine-like (Sc/NE) and Mesenchymal-like (Mes-like) [8]. We and others have observed subtype intratumor heterogeneity (ITH) in primary tumors and between bladder tumors and matched lymph-node metastases [9–13]. However, the frequency of subtype ITH in MIBC is debated and depends on how subtypes and ITH are defined. ITH may arise from cellular plasticity, where cancer cells transition between phenotypic states [14, 15] or through stable coexisting subpopulations during tumor evolution [16, 17]. Ultimately, this heterogeneity may lead to genomic differences between primary tumors and distant metastases [18], potentially limiting the reliability of molecular profiling from the primary tumor in guiding systemic therapy. We hypothesize that some tumors or subtypes may be more dynamic and could change the cancer cell phenotype in response to changes in the microenvironment. Notably, significant differences in the tumor microenvironment (TME) have been observed between primary tumors and metastases [19]. While research on the TME in bladder cancer [20] remains limited compared to other cancers [21], key components such as cancer-associated fibroblasts, immune cells, vasculature, cytokines, and extracellular matrix (ECM) proteins could influence bladder cancer biology [22]. Similarly, in the metastatic TME, different ECM compositions, oxygen levels, tissue-resident fibroblasts, and tissue stiffnesses may also drive phenotypic changes and divergent evolutionary trajectories in metastatic tumor clones. Specifically, we hypothesize that the luminal phenotype of urothelial-like bladder cancer, regulated by GATA3 and PPARG, may be modulated by the microenvironment, similar to the findings in breast cancer models where intraductal implantation preserves luminal differentiation, whereas fat pad implantation induces a basal-like state [23]. The basal and luminal subtypes of bladder cancer share molecular features with basal and luminal breast cancers, including the TP63 activation in the basal tumors and overexpression of PPARG in the luminal tumors.

For this study, we developed and characterized seven patient-derived bladder cancer xenograft (PDX) models, including five with spontaneous metastatic potential. These models, which represent the three most common BC subtypes (Uro, GU, BaSq), were propagated in mice in different microenvironment niches: mesenchymal-rich (subcutaneous), adipocyte-rich (mammary fat pad), and epithelial-rich (subrenal capsule) to assess the stability of discrete molecular phenotypic states. Subrenal capsule transplantation has been shown to increase PDX engraftment rates, particularly for less aggressive tumors, by providing a highly vascularized environment with abundant nutrients, oxygen, and growth factors, as well as facilitating metastatic dissemination [24, 25]. In contrast, the subcutaneous space, which is widely used because of its accessibility and ease of monitoring, lacks these supportive characteristics. The mammary fat pad presents a complex microenvironment composed of adipocytes, fibroblasts, and endothelial and immune cells. The adipocytes and stroma in the fat pad secrete factors such as PGE, hepatocyte growth factor, heregulin, IGF, FGF1/7, TGFα, and Wnt, which promote epithelial cell growth, tumor progression, metabolism and invasion [26–31]. Some of these factors have also been linked to BC progression and prognosis [32–34].

We applied comprehensive multiomics analyses, including exome and RNA sequencing, and protein expression data to test whether BC molecular subtypes maintain or change their identity in response to these three distinct anatomic locations. We also tracked the forward genomic evolution of these established MIBCs to separate patterns of adaptation from ITH related to clonal evolution. Finally, we compared subcutaneous tumors with tumor subpopulations arising from spontaneous and experimentally induced metastasis to assess genetic stability and phenotypic plasticity during tumor progression and metastasis. As bladder cancer treatment advances towards precision medicine and personalized therapies, understanding these dynamics is crucial for optimizing therapeutic strategies [35].

## Methods

### PDX selection and experimental setup

Transcriptomic and genomic data from bladder cancer PDX models available through The Jackson Laboratory were analyzed to identify models representative of three main LundTax molecular subtypes: Urothelial-like (Uro.1-2), Genomically Unstable (GU.1-2) and Basal/Squamous-like (BaSq.1-4). The data and information for the PDX models are publicly available from the JAX PDX portal database (http://tumor.informatics.jax.org/mtbwi/pdxSearch.do) and are summarized in Supplementary Table 1. Uro and GU tumors have relatively well-defined genomic profiles enriched for the loss of *CDKN2A* and *RB1*, respectively. In contrast, BaSq tumors lack a defining mutational or genomic profile and are also more aggressive and treatment resistant [7, 36]. To capture this diversity, we selected two BaSq models with *CDKN2A* loss and two models with *RB1* loss. We generated models from passage 3–4 and all experiments were carried out within the first 6–9 passages after the tumor models arrived at our laboratory. We used 5- to 7-week-old female NOD-*scid* IL2Rg^null^ (NSG) mice maintained under pathogen-free conditions.

After initial subcutaneous (SC) propagation to expand and cryopreserve the models, the tumors were transplanted to the mammary fat pad (FP) and under the kidney capsule (KD) and passaged for three additional generations in parallel with SC propagation (see Supplementary Fig. 1 for details). Tumors from these three locations are defined as the primary tumors in the current study. Tumor inoculation was performed using 1–2 mm³ tissue fragments, implanted through small incisions for SC (*n* = 3–5 per passage, with bilateral inoculation in the flanks) and FP (*n* = 3 per passage) models, and after kidney exposure for KD (*n* = 3 per passage) models. One of the Uro models (Uro.2) did not grow when transplanted to new mice and was excluded from the study. In selected SC and FP models the implanted tumor was surgically removed once it reached 1–1.2 cm in diameter, the incision was sutured, and the mice were monitored for up to two additional months or until signs of declining health to allow metastasis development. For the experimental metastasis assay, fresh tumor samples from one of the three anatomic sites were dissociated, and tumor cells suspended in PBS were injected into the tail vein or left ventricle to induce metastasis (*n* = 3–6). If no metastases developed within six months, the number of injected cells was increased from 10,000 to 50,000 and 100,000 in subsequent experiments in a new group of mice.

For each model, samples from the first and third generations of tumors in each anatomical location were processed for RNA and DNA analysis. In the case of the SC tumors, additional samples were processed during expansion, including tumors from different mice in the same passage, as well as from the latest passages. All macrometastases, defined as those visible to the naked eye, were sampled for RNA and DNA extraction and formalin-fixed for downstream analysis. The lungs and any organ with suspected findings were formalin-fixed and processed for histological evaluation. Metastases screening was routinely performed for all inoculated mice during necropsy and using one H&E-stained section of the lungs.

### Tumor sample collection and processing

Tumor samples and murine lungs were harvested and fixed in 10% neutral-buffered formalin for histological analysis. For DNA and RNA analysis, non-necrotic tumor fragments, carefully dissected to minimize contamination with surrounding murine tissue, were collected in Allprotect tissue reagent and processed for nucleic acid extraction as described in the Supplementary Materials. A total of 525 samples were selected for tissue microarray (TMA) construction and immunohistochemistry (IHC) staining, 97% of which were suitable for evaluation. RNA-seq, exome sequencing, and SNP array analyses were carried out on 120 unique samples. One exome-sequencing sample and one RNA-sequencing sample showed insufficient coverage, as specified below, and were excluded from analysis.

### RNA sequencing and data analysis

Library preparation and RNA sequencing were performed by SciLifeLab in Lund. Total RNA (500 ng) was barcoded and prepared for sequencing via the TruSeq Stranded mRNA Library Prep (20020594, Illumina). Libraries were amplified with 13 cycles of PCR, pooled and sequenced on a NovaSeq 6000 (20012850, Illumina) using SP (32 + 32 samples) or S1 (64 samples) flow cells with 100-bp or 150-bp paired-end reads, targeting a mean of 40 million read pairs per sample.

FASTQ files were preprocessed and aligned via the nf-core/rnaseq pipeline (v. 3.9) using *nextflow* (v. 22.04.5) as detailed in Supplementary Methods. Gene IDs were annotated using the Ensembl *biomaRt* R package (v. 2.56.1) with the hsapiens gene Ensembl dataset (v. GRCh38.13) to obtain gene symbols and biotypes. Downstream analysis was performed using protein-coding and non-mitochondrial genes. Tumors were classified into molecular subtypes using the LundTax2023 random forest rule-based single-sample predictor [8]. RSEM merged gene counts were used to identify differentially expressed genes (DEGs) with the *DESeq2* R package (v. 1.38.3) as detailed in Supplementary Methods. Cluster analysis and visualizations were conducted in R (v. 4.2.3) using Variance Stabilizing Transformation (VST) and Principal Component Analysis (PCA) functions from *DESeq2* for data normalization and dimensionality reduction. *Rtsne* (v. 0.16) was used for t-distributed stochastic neighbor embedding (t-SNE). The specific parameters used in each analysis are detailed in the respective figure legends. Hierarchical clustering and dendrograms were generated based on the 500 top-varying genes using Pearson’s correlation coefficient and Ward’s method.

Functional gene annotation was conducted using the R packages *clusterProfiler* (v. 4.8.1) and *fgsea* (v. 1.26.0) for individual models and differentially expressed genes across tumor locations. Multiple testing was controlled using Holm’s method for family-wise error rate (FWER) correction within each location. Relevant gene signatures for gene set enrichment analysis were downloaded from https://www.gsea-msigdb.org. Downstream ontology analysis focused on terms present in more than one model and supported by at least three DEGs. Functional grouping of terms obtained from different models was performed using the *enrichplot* package (v. 1.20.0).

### Exome sequencing and analysis

Library preparation and sequencing were performed by the SNP&SEQ Technology Platform at Uppsala University. Sequencing libraries were prepared from 50 ng DNA using the Twist Comprehensive Exome sample preparation kit and probe panel (cat# 101897/102031 or 101901/102032, Twist Bioscience) with unique dual indices. Library preparation was performed according to the manufacturer’s instructions (DOC-001085). Exome libraries were sequenced on a NovaSeq 6000 system, S4 flow cell and v1.5 sequencing chemistry at a 2 × 150 bp read length. A total of 120 samples were sequenced in 3 batches (45, 45, 30).

The raw fastq files were preprocessed using the *Xenome* package [37] with default parameters to separate human reads from mouse reads, with Human hg38 and Mouse mm39 as reference genomes. Only the human reads were used for downstream analysis. The filtered fastq files were preprocessed with the Sarek pipeline [38] using *Nextflow* (v. 22.04.5, nf-core/sarek: v. 3.0.1), which includes quality control and trimming, mapping, duplicate read identification, and base quality score recalibration based on GATK4 best practices. *Mutect2* (v. 4.2.6.1) and *Strelka* (v. 2.9.10) were used for variant calling in a tumor-only setting through the nf-core/sarek pipeline. Sequencing coverage was calculated with *Mosdepth* (v. 0.3.3). A mean coverage greater than 20X across coding regions was set as the threshold to retain samples for downstream analysis. Called variants were annotated using Ensembl Variant Effect Predictor (*VEP*) (v. 108_GRCh38). In addition to the quality and technical filters applied by both *Mutect2* and *Strelka*, a germline filter was applied by Mutect2-based variant frequency on 1000G/panel of normals and gnomAD databases and a likely germline algorithm. The union of “PASS” variants from either caller was further filtered to exclude (a) germline variants (allele variants occurring in more than 1% of the population, according to *Strelka* and *Mutect2* annotations), and (b) artifacts (either variants with coverage less than 10X in any of the samples of a given model, variants with mean allele frequency less than 5%, or variants with alternative allelic depth less than 10). When identifying private variants, a minimum locus depth of 20 was used.

The tumor mutational burden (TMB) was calculated as the total number of somatic, coding, base substitution, and indel mutations per megabase of the genome covered by at least 10 reads.

A list of annotated cancer genes was obtained from the cosmic (COSMIC v97, released 29-NOV-22) and OncoKB databases (update 02/01/2023). A list of cancer hotspots was obtained from https://www.cancerhotspots.org/, version V2.

### SNP array and chromosome alteration analysis

Genotyping was performed using the Illumina Infinium Global Screening Array-24 (GSA-MD) v3.0, and the results were analyzed using the software *GenomeStudio* (v. 2.0.3) by the SNP&SEQ Technology Platform at Uppsala University. A total of 120 samples were analyzed in 3 batches (45, 45, and 30) using the GRCh38 reference genome. SNP array data from PenCNV files were processed in R to extract probe, segment, and SNP information for normalization and segmentation using *TAPS* (v.1.9) [39]. A detailed description of the methodology used for segment estimation and annotation is provided in Supplementary Methods.

Segments ≥ 0.1 Mb in length were included in downstream phylogenetic analyses. Copy number alterations (CNAs), identified based on relative coverage ratios and allelic imbalances, were classified as clonal or subclonal according to their distribution in SNP array-based estimates. Subclonal deconvolution was performed using the *DEVOLUTION* algorithm (v. 1.1) to resolve multiple subclones within each sample, where present [40]. Detailed information for each CNA—including genomic coordinates, alteration type (e.g., gain, loss, or copy number-neutral imbalance), and the proportion of cancer cells harboring the alteration (mutated clone fraction, MCF)—was curated and compiled into a segment file. An event matrix was generated from each dataset and used for phylogenetic reconstruction, employing both maximum parsimony and maximum likelihood methods via the *phangorn* R package (v. 2.12.1) [41]. The resulting phylogenies were visualized using *ggplot2* (v. 3.5.1) in R. MCF values, serving as a proxy for clone size, were calculated for all CNAs under the assumption of a balanced diploid baseline. Aberrations with MCF ≥ 85% were considered clonal, consistent with previous studies applying subjective thresholds. This 85% cutoff was determined post hoc due to the high purity of bulk-sequenced samples. For detailed phylogenetic analysis methodology, see Supplementary Methods.

### Histology and immunohistochemistry analysis

Sections from tumors, lungs, and other organs with suspected metastasis were evaluated via Hematoxylin and Eosin (H&E) staining for histological assessment and to select areas for TMA construction. The TMAs included two 1 mm formalin-fixed, paraffin-embedded tissue cores from tumor-rich areas of all tumor blocks and other tissues with macrometastases. TMA sections were stained for 13 bladder cancer-specific markers: CCNB1, CK5, CK14, RB1, PPARG, GATA3, FGFR3, EGFR, CDH3, CCND1, P63, E2F3, CDKN2A(p16), CK20, UPK2, and ASMA on the automated DISCOVERY ULTRA instrument (Ventana Medical Systems Inc, Tuscon, AZ) using DAB for detection. Details on the antibodies are provided in Supplementary Table 2. To avoid cross-reactivity, IHC staining with mouse antibodies included an additional incubation step with a rabbit monoclonal anti-mouse IgG antibody (ab133469, Abcam). Tumor cells expression was evaluated as the proportion of positive cells (score 0–5) and/or intensity of staining (score 0–3). Tumor cell scores were calculated by multiplying the staining proportion by the intensity. The staining pattern was evaluated for CK5 (negative, few, diffuse, noncontinuous basal cell staining, multilayer, all) and for EGFR, CDH3, and CCNB1 as stratified or diffuse staining as previously defined [42]. Markers with greater spread (interquartile range or standard deviation > 0.5) were tested to identify differentially expressed markers between groups using ANOVA and two-sided unpaired t tests followed by Bonferroni correction.

## Results

**Fig. 1:**
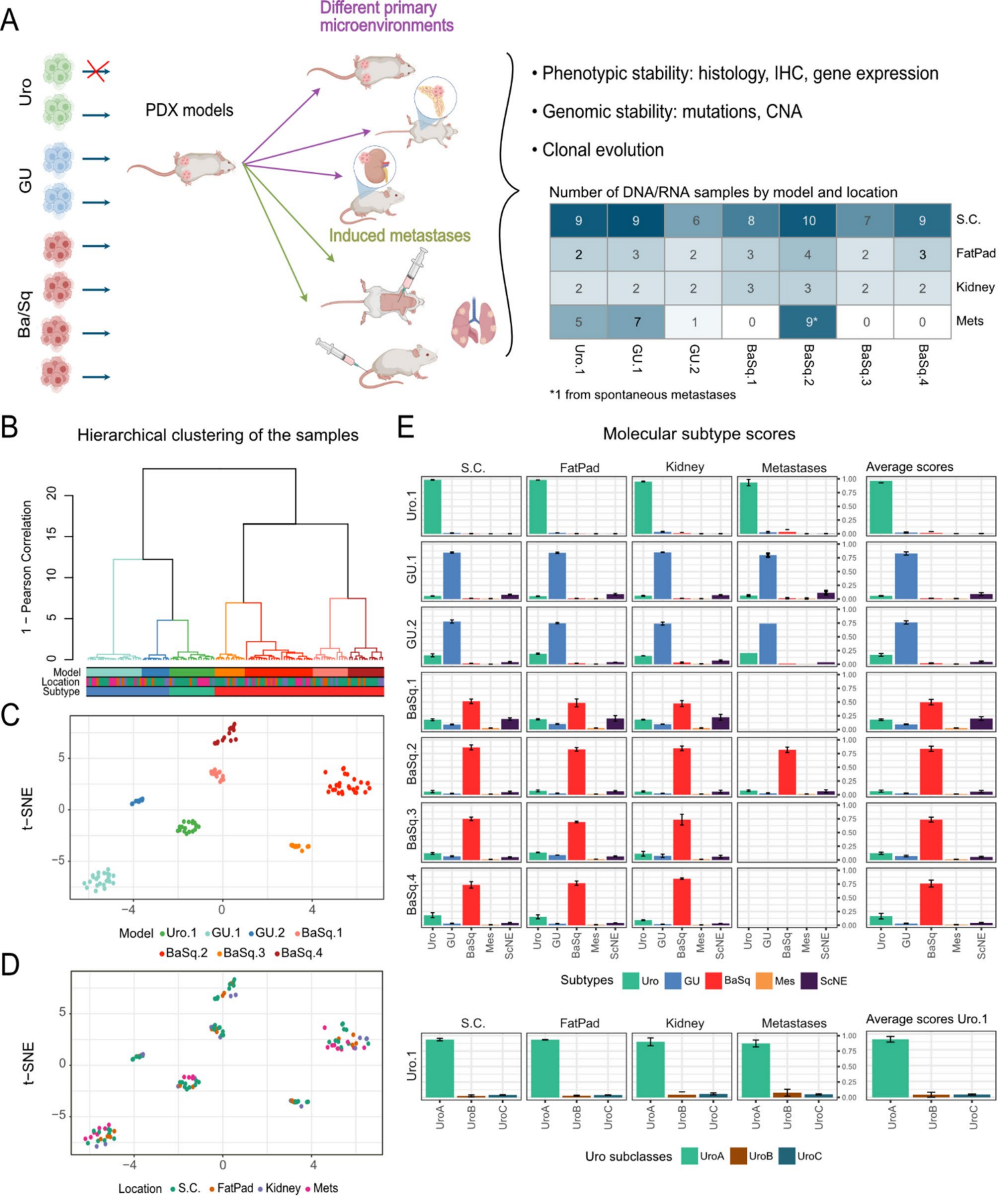
Study design and cohort characterization using RNA data for samples clustering and molecular subtyping. Eight PDX models representative of the three main LundTax molecular subtypes — Urothelial-like (Uro.1), Genomically Unstable (GU.1-2), and Basal/Squamous-like (BaSq.1-4) — were selected for this study. Seven models were successfully propagated into three anatomic locations: subcutaneous (SC), mammary fat pad (FP) and under the kidney capsule (KD). Both spontaneous metastases and metastases induced via intracardiac or tail vein injection were also analyzed. One Uro PDX model (Uro.2) failed to grow after subcutaneous passaging and was excluded from further analysis. Selected samples from primary tumors and experimentally induced metastases, along with one spontaneous lung metastasis, were processed for RNA and DNA extraction for exome sequencing, SNP array, and RNA sequencing (**A**). Hierarchical clustering of a Pearson correlation matrix based on the top 500 most variable genes in all models. Branches are colored by model and colored bars indicate the model, location and subtype of the samples (**B**). Clustering of the samples based on the t-distributed stochastic neighbor embedding (t-SNE) method annotated by model (**C**) and location of the samples (**D**). Bar plots illustrating the average molecular subtype scores for each location and the global average per model (**E**)

To study the propensity of bladder cancer to undergo microenvironment-mediated tumor plasticity and genomic and phenotypic adaptations, we generated and characterized seven PDX models representative of the Urothelial-like (Uro.1), Genomically Unstable (GU.1-2) and Basal/Squamous-like (BaSq.1-4) molecular subtypes. Tumors were challenged by inoculation into three anatomic locations: subcutaneous (SC), the mammary fat pad (FP), and under the kidney capsule (KD). All models that formed SC tumors also formed tumors in the FP and KD groups. Between 2 and 11 tumors per model and anatomic site were sampled for RNA sequencing and genomic analysis (Fig. 1A, Supplementary Fig. 1).

### BC PDX models with intrinsic capacity to form spontaneous and induced metastases

Spontaneous metastases developed in 5 of the seven models (GU.1, GU.2, BaSq.2, BaSq.3, and Uro.1) from tumors at the SC, FP, or KD sites, with variations in lesion size and frequency across the models. With the exception of the BaSq.2 model, spontaneous metastases were often too small for identification during necropsy and to allow for efficient DNA/RNA extraction from fresh tissue. To systematically profile the metastases, up to 100,000 dissociated SC tumor cells were injected into the tail vein or into the hearts of NSG mice. Metastases developed in the same five models that exhibited spontaneous metastases, suggesting that metastatic potential is tumor intrinsic and depends mainly on the later steps of the metastatic cascade (Supplementary Table 3). Spontaneous metastases were restricted to the lungs, whereas induced metastases were larger and disseminated to multiple organs, including the lungs, liver, adrenal glands, ovaries, and pancreas (Supplementary Tables 3 and Supplementary Fig. 2). The BaSq.2 model was highly metastatic, frequently forming multiple lung macrometastases from primary tumors (i.e. from the three primary implantation sites), and all animals developed metastases following injection, predominantly to the lungs. In contrast, spontaneous lung metastases in the Uro.1 and GU.1 models were less common and typically microscopic, with induced metastasis rates of 38% and 29%, respectively. GU.2 and BaSq.3 displayed low metastatic potential, with only 10% of the injected mice developing small lung lesions and rare cases of spontaneous metastases.

### Transcriptomic profile and morphology remain stable across environments and in metastatic sites

To test the influence of the microenvironment on the tumor phenotype, we performed hierarchical clustering on the RNA-seq data, which revealed a distinct division between luminal (Uro and GU) and non-luminal (BaSq) samples (Fig. 1B). At the seven-cluster level, the branches corresponded completely with the models. The high degree of similarity within each model was confirmed by t-SNE, which revealed clusters separated by model and not by location (Fig. 1C-D, Supplementary Fig. 3). To identify subtype shifts during propagation or metastasis, we applied the LundTax single-sample predictor, which assigns a score for each molecular subtype. Subtype instability or emerging intratumor heterogeneity (ITH) would be indicated by variations in these scores. However, no such changes were observed, as each model presented nearly identical score profiles across all samples, regardless of the microenvironment (Fig. 1E, Supplementary Figs. 4 and 5).

**Fig. 2:**
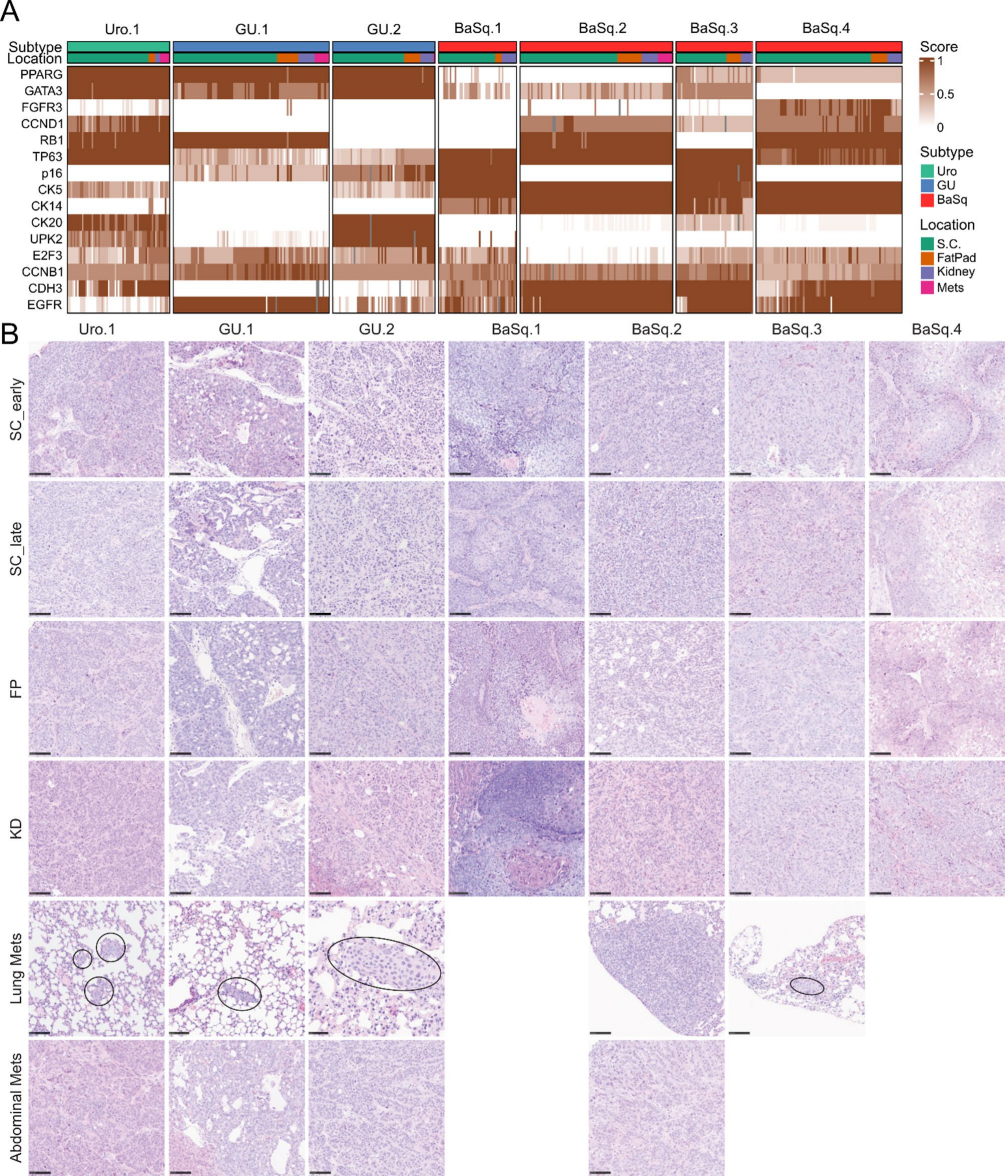
Immunohistochemical and histological characteristics of the tumors. Heatmap based on the normalized immunohistochemical expression levels for the indicated markers evaluated as intensity, the proportion of positive cells, or the tumor cell score (intensity*proportion). Grey bars indicate missing values. Within each model, samples were sorted by location and passage number (**A**). The Uro.1 model was characterized by high levels of differentiation markers (GATA3, PPARG, CK20, and UPK2) as well as TP63 and CDH3. This model was consistently negative for p16 and FGFR3, with low expression of CK5, CK14, and EGFR. The GU.1 tumors were positive for PPARG, GATA3, EGFR, and RB1 but negative for CKs, UPK2, and CDH3 whereas the GU.2 tumors were positive for PPARG, GATA3, CK20, and UPK2, but negative for RB1, coupled with intermediate to very high p16 expression. Both models were negative for FGFR3 and CCND1. The staining patterns were consistent across locations, except for minor increases in UPK2 expression in GU.1 in the FP samples. All basal models exhibited extensive expression of CK5/14, TP63, and EGFR, with intermediate CDH3 expression. BaSq.4 was FGFR3 positive, and BaSq.2/3 presented low expression levels of GATA3 and PPARG. The staining patterns remained stable across locations, with minor changes, such as decreased CK14 in kidney tumors of BaSq.3 and increased CCNB1 in BaSq.4. Representative images of tumor morphology at different passages and locations (subcutaneous (SC), fat pad (FP), under the kidney capsule (KD), lung and abdominal metastases) (**B**). The images were obtained at 20X magnification, and the scale bar indicates 100 μm. Micro lung metastases are indicated by black circles. SC_early and SC_late indicate samples from early and late passages, respectively

To assess any emerging subtype of ITH in the form of a minor subpopulation of cells potentially missed by bulk RNA-sequencing, we analyzed immunohistochemical staining patterns with subtype-defining protein markers (Fig. 2A, Supplementary Figs. 6 and 7). The Uro.1 model was characterized by solid tumors growing in large nests with palisading cells around a fibrovascular core. While robust urothelial basal-cell stratification (CK5/EGFR/CDH3) typical of Uro tumors was absent, scattered CK5-positive cells were localized at the tumor-stroma interface (Supplementary Fig. 6), which is how more aggressive Urothelial-like tumors typically present. The two GU models were quite distinct: GU.1 was fast-growing, often developing cysts and forming highly vascularized and invasive tumors, whereas GU.2 was slow-growing and encapsulated with cells organized in small nests. The four BaSq models tended to be either slow-growing with keratinization, encapsulation, and limited invasiveness (BaSq.1/4) or fast-growing and more invasive (BaSq.2/3). These differences were not explained by the RB1/p16 status as BaSq.1/3 were RB1- and BaSq.2/4 were p16-. Over time, markers related to canonical oncogenes or tumor suppressors (RB1, p16, and FGFR3) were stable, whereas some proliferation (CCNB1, E2F3, and CCND1) and differentiation markers (CDH3, CK5, CK20, EGFR, and TP63) fluctuated slightly (Supplementary Figs. 7 and 8). However, these differences reflect stochastic variation rather than significant trends across anatomic sites. Similarly, growth patterns, histological features and stromal content varied by model but remained consistent over time and across the different locations, including in metastases (Fig. 2B). In summary, the immunohistochemical analysis did not reveal any signs of focal subtype ITH in the seven models. The morphology, growth patterns, and key molecular markers were maintained during prolonged passaging across different microenvironments and in metastatic lesions.

### Differentially expressed genes between anatomic locations and metastasis

**Fig. 3:**
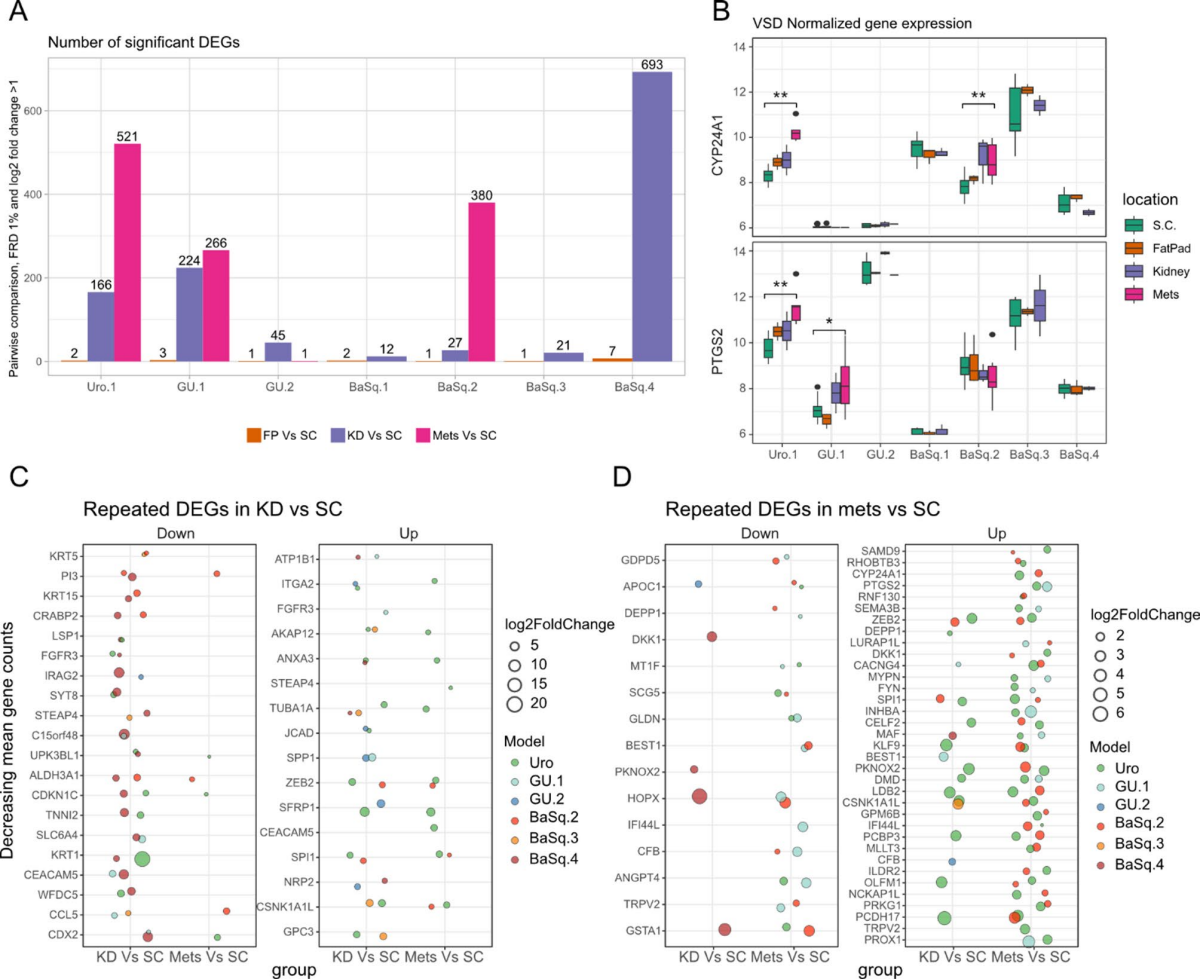
Differential expression analysis. The number of differentially expressed genes (DEGs) across models and different locations after adjusting for a false discovery rate of 1% and filtering for genes with an absolute log2-fold change greater than 1 (**A**). Boxplots showing the relative gene expression level of *CYP24A1* and *PTGS2* within models and across locations. Statistical analysis was performed using one-way ANOVA to assess overall group differences, followed by pairwise t-tests for post hoc comparisons. Asterisks indicate significance from the pairwise tests: *p* < 0.05 (*), *p* < 0.01 (**). No correction for multiple comparisons was applied (**B**). Dot plot showing up- and downregulated DEGs shared in at least 2 models when comparing tumors grown under the kidney capsule vs. subcutaneous (SC) tumors (**C**) or between metastases and SC tumors (**D**). The size of the dots represents the log2-fold change for a given gene, and the color reflects the model where the genes were found to be differentially expressed

To further investigate potential mechanisms of tumor adaptation to microenvironments, we analyzed differentially expressed genes (DEGs) across tumor sites within each model and identified shared genes and biological processes. We compared tumors from the FP, KD, and metastases with SC tumors. SC and FP tumors presented similar transcriptomes, with only 1–7 significant DEGs, whereas KD tumors and metastases presented greater transcriptional divergence from SC tumors, with 12–693 DEGs in KD and 266–521 DEGs in metastases (FDR < 1%, log2FC > 1) (Fig. 3A, Supplementary Fig. 9). Among the metastasis-associated DEGs with the highest expression levels, *CYP24A1* and *PTGS2* were significantly upregulated in metastases, with *CYP24A1* elevated in the Uro.1 and the BaSq.2 models, and *PTGS2* in Uro.1 the GU.1 models, respectively (Fig. 3B). The DEGs were not particularly associated with kidney, lung, or mammary fat pad cell types. Some of the DEGs were shared between the models: 33 (2.9%) from KD vs. SC and 43 (3.8%) from metastases vs. SC with and between groups (Fig. 3C and D). While most shared DEGs were not subtype specific, epidermal keratins (e.g., *KRT1/5/15*) were downregulated in KD vs. SC in the BaSq.2–4 models, consistent with the reduced keratinization and epidermis-related GO term analysis for the same models (Supplementary Fig. 10). In BaSq.3, but not in BaSq.2/4, the expression of basal keratins (CK5/14) also decreased at the protein level. *FGFR3* is an important oncogenic driver in BC, and its expression was decreased in KD tumors from the BaSq.4 and Uro.1 models but upregulated in GU.1 tumors compared with that in SC tumors. Concordant changes in FGFR3 protein expression were observed only in BaSq.4, which had a clonal *FGFR3* S249C mutation. Hypoxia-related genes (*STEAP4*,* ALDH3A1*,* ANX3*, and *SPP1*) were among the shared upregulated genes in the KD vs. SC comparison, potentially reflecting an adaptation to oxygen availability. The DEGs with higher expression were associated with inflammation and tissue remodeling (PI3) and adhesion/invasion (*ITGA2*,* AKAP12*,* ANXA3*,* JCAD*,* SPP1*, and *ZEB2*) most of which also increased in metastases samples. Accordingly, epithelial-to-mesenchymal transition, ECM-receptor interaction, and focal adhesion were among the few significant terms in the ontology analysis, albeit not in a consistent way (Supplementary Fig. 10).

In the metastasis group, the top shared upregulated genes were cancer-related with diverse functions, including tumor suppressors (*SAMD9* and *SEMA3B*) and genes involved in growth, survival, cytoskeletal dynamics, motility, invasion, and metastasis (*RHOBTB3*,* CYP24A1* and *PTGS2*). Metastatic samples from all three molecular subtypes presented a fivefold increase in *CYP24A1* expression, encoding vitamin D-24-hydroxylase, the key negative regulator of calcitriol (active form of vitamin D). Calcitriol can suppress PGE_2_ (prostaglandin E2) production by inhibiting COX-2 (*PTGS2*), which was also upregulated in metastases (Fig. 3B **and D**). Pathways and terms related to EMT, interferon alpha/gamma response, and TNFA signaling were found to be significant in individual models but inconsistent between models (Supplementary Fig. 11). Approximately half of the shared DEGs identified in metastases were also present in the KD vs. SC comparisons, suggesting that some changes, such as ZEB2 upregulation in Uro.1 and BaSq.2, were not exclusive to metastases (Fig. 3D). These findings highlight tumor-specific and shared transcriptional adaptations during growth in different microenvironments and metastasis.

### Gene mutation profiles remain stable

**Fig. 4:**
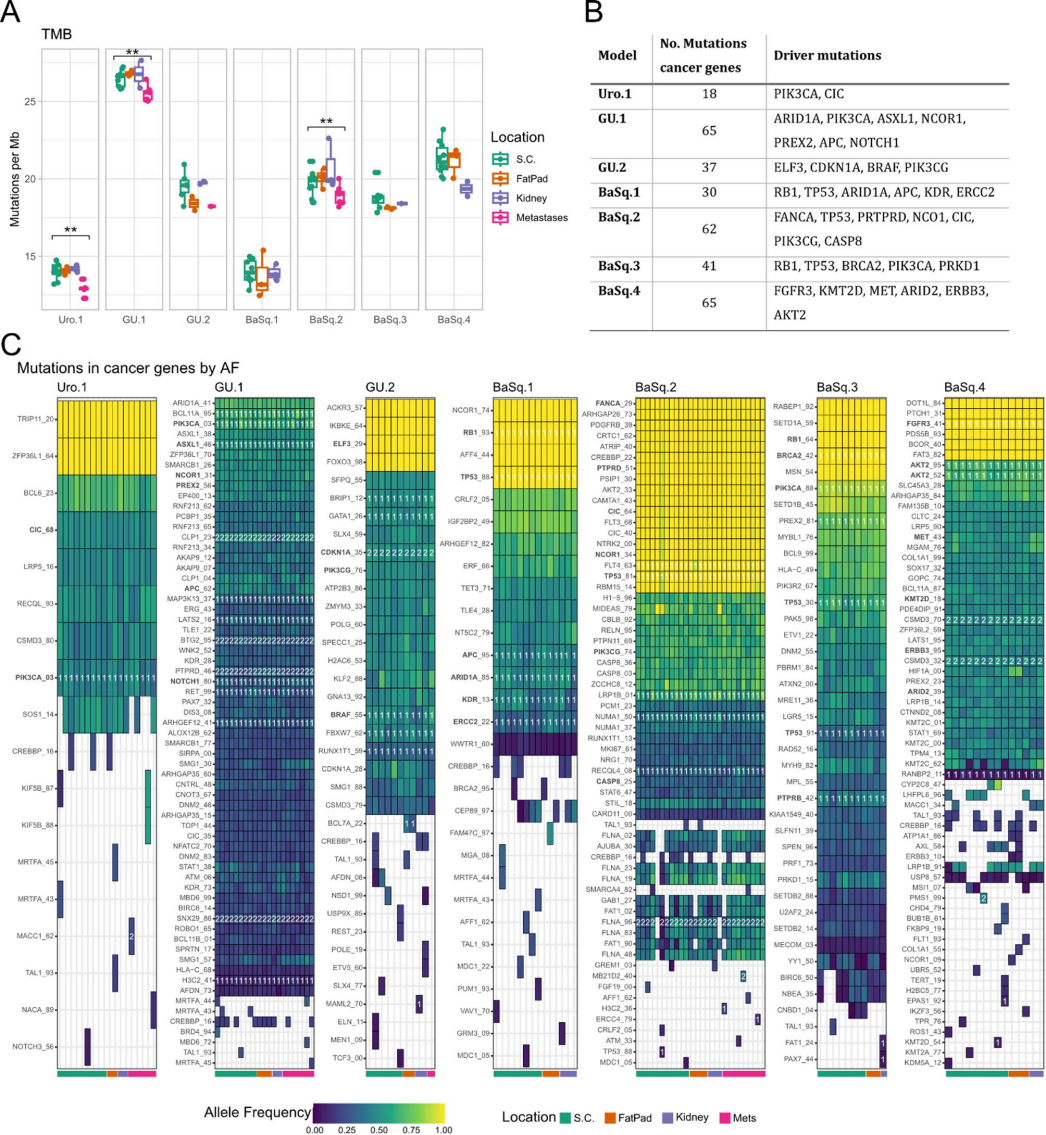
Tumor mutational burden (TMB) and mutation profile of samples from different environments and over time. Boxplot showing the estimated TMB stratified by location across the different models (**A**). Statistical analysis was performed using one-way ANOVA to assess overall group differences, followed by pairwise t-tests for post hoc comparisons. Asterisks indicate significance from the pairwise tests: *p* < 0.01 (**). No correction for multiple comparisons was applied. The number of mutations in cancer-related genes and potential drivers in each model (**B**). Heatmaps showing the cancer-related mutations present in each sample across the different models (**C**). The colors reflect the allele frequency of a given event or white when the mutation was absent. In the case of GU.1 model, none of the variants had an allele frequency of 1 due to polyploidy. The colored bars indicate the location. The samples are sorted by location and passage number. Potential driver mutations are marked in bold, and the numbers after the gene name indicate the genomic position, allowing the identification of distinct variants affecting different alleles of the same gene, for example, *TP53* in the BasSq.3 model

We analyzed the tumor mutational burden (TMB) and mutational profiles across the models and growth environments. Nonsynonymous TMB ranged from 13.76 to 27.65 mutations/megabase, with the highest values in the GU.1 model and the lowest in Uro.1 and BaSq.1 (Fig. 4A). The relatively high TMB, despite stringent filtering, suggests that a fraction of germline variants may remain in the dataset. This is a known limitation in the absence of matched normal samples and should be considered when interpreting absolute TMB values and the proportion of private mutations. The TMB was consistent across different growth sites, with a significant decrease in metastases in 3 models. This decrease might reflect the loss of certain clonal populations during the metastatic process. Tumor cell dissociation, survival in circulation, and the successful colonization of distant organs all impose selective pressures that may lead to a genetic bottleneck, reducing overall mutational diversity.

Mutations were identified in 2,854 genes across all models, with 9–17% of mutated genes in each model corresponding to cancer-related genes (COSMIC and OncoKB databases). Analysis of cancer-related mutations excluded hypermutated genes such as *MUC4* and revealed 18–65 mutations per model, which was largely consistent across samples (Fig. 4B-C). We considered COSMIC census tiers, variant impact, allele frequency, gene expression level, and frequency in bladder cancer (TCGA) to identify potential driver mutations (Fig. 4B).

Private variants within tumor models accounted for 0.29–2.62% of the total number of mutations, which was comparable to the interbatch variability (2.38%). This suggests that most private variants were likely artifacts, whereas low-frequency variants shared across some of the samples reflected regional differences within the tumors. Only a few *de novo* variants were passed on downstream; one example was an *AXL* mutation (p.A681L) in BaSq.4, detected at 15% allele frequency in a passage 3 subcutaneous tumor and later increasing to 25–37% in subsequent samples as well as in an FP sample that was derived from an upstream sample (p2). Another mutation was a deleterious *BCL7A* mutation (p.S186C) in GU.2, present only in fat pad tumors with 39 and 26% allele frequency. One SNV in *CREBBP* was observed at a variable low frequency across five models. This mutation is a potential mouse-derived artifact and should be interpreted with caution.

Overall, mutational landscapes, including TMB and profiles, were stable across different tumor microenvironments, indicating minimal genomic adaptation during environmental transitions.

### Copy number alterations (CNAs)

**Fig. 5:**
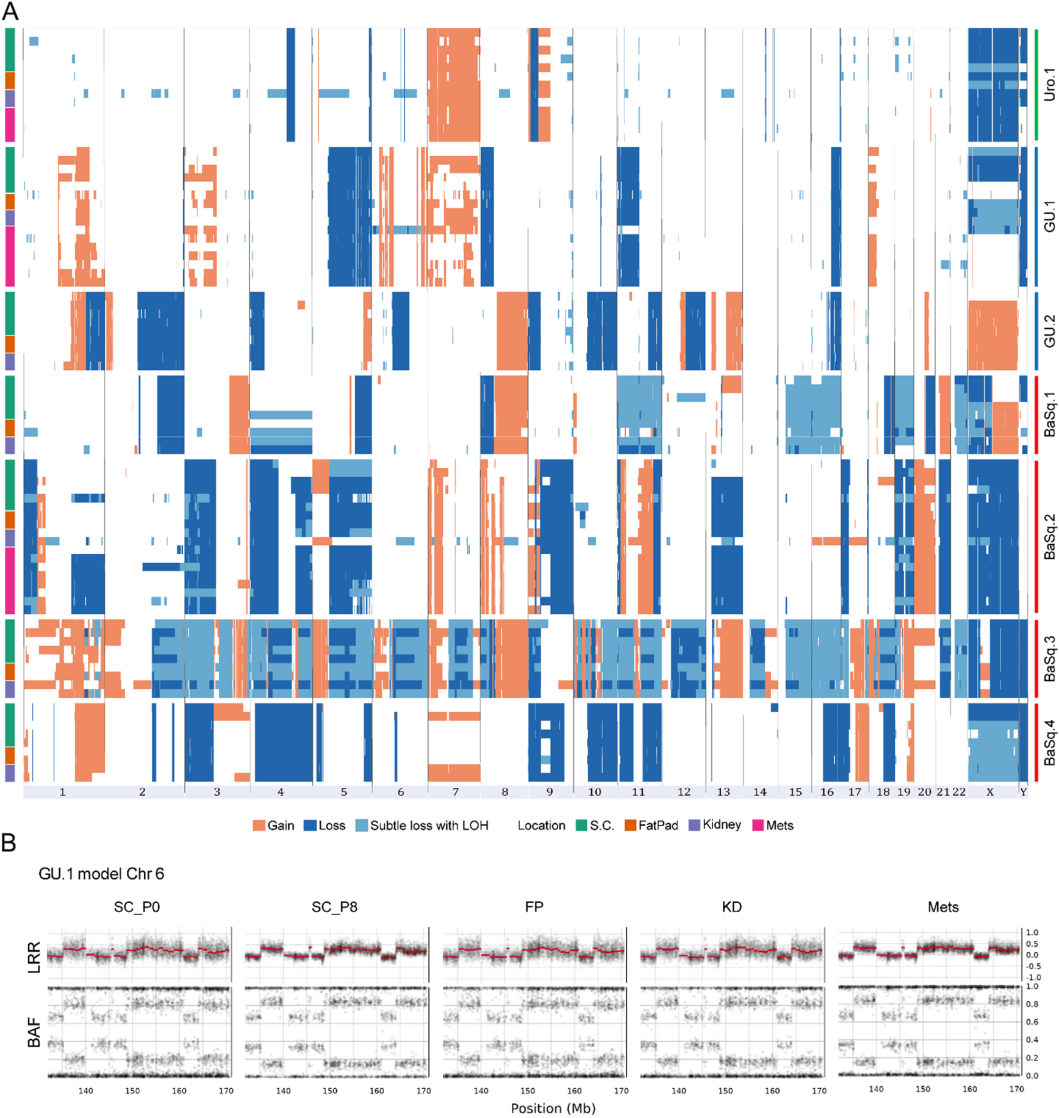
Copy number profiles within each model across locations. Heatmap of copy number alterations across chromosomes grouped by model and location with samples sorted by location and passage number (**A**). Log R ratio and B allele frequency plots showing an example of a complex chromosome rearrangement in chromosome 6 observed across all samples of the GU.1 model (**B**)

**Fig. 6:**
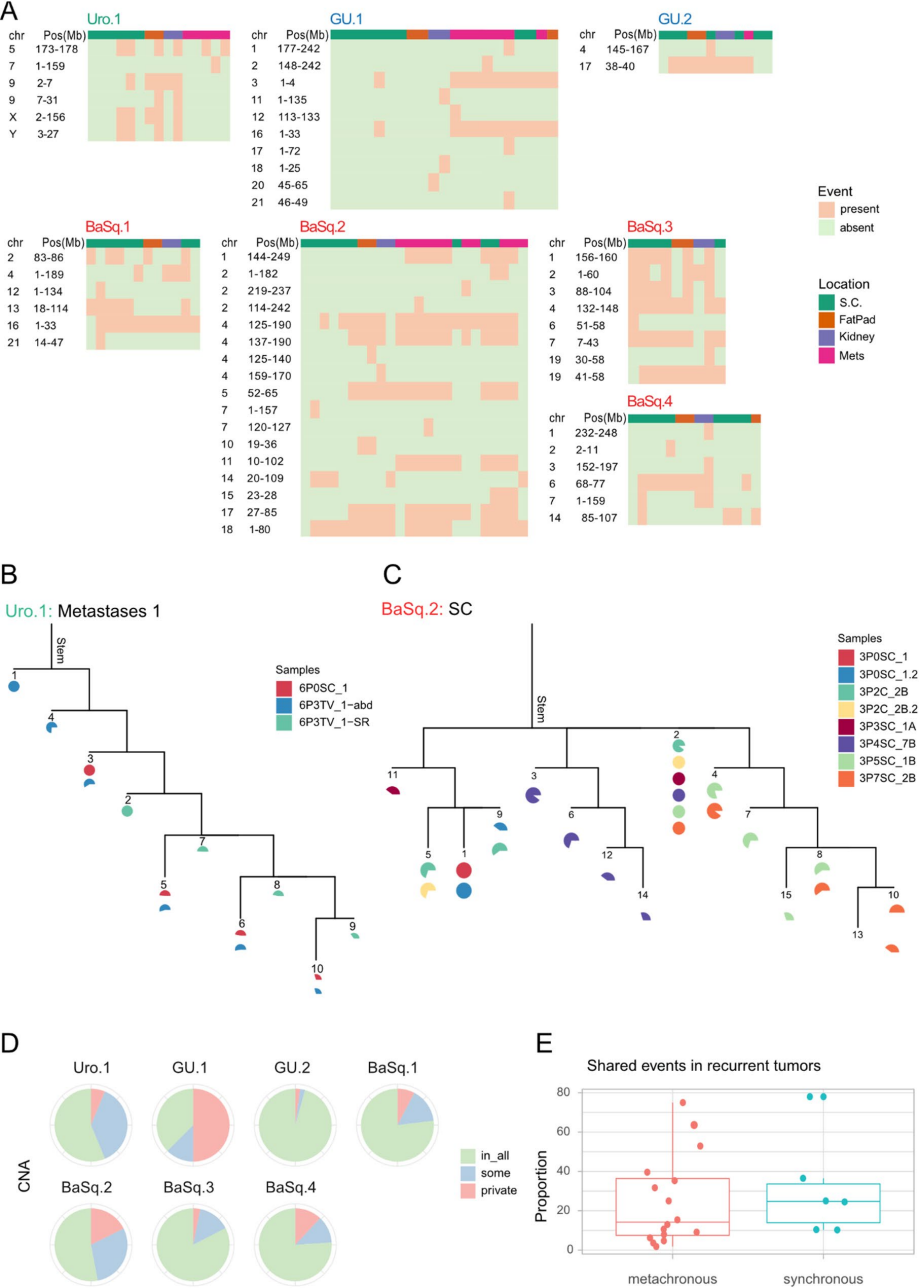
Profile of new chromosomal alterations observed during tumor propagation. Heatmaps showing the pattern of CNA alterations that were present in some samples but not all samples in each model. Within each model, samples were sorted by location and passage number (**A**). Phylogenetic trees of a metastatic lineage in the Uro.1 model (**B**) and the subcutaneous lineage in the BaSq.2 model (**C**) illustrating a case of linear evolution and complex branching evolution, respectively. At the top of the tree is the stem, which represents the genetic alterations shared by all cells across all biopsies. Adjacent to the stem, the biopsies included in the tree are depicted as filled pie charts in different colors. The entities at the leaf nodes correspond to distinct groups of cells with unique genomic profiles (subclones), labeled with numbers. The branches indicate in which biopsies the subclones are found and their relative proportions within those biopsies. Pie chart showing the global proportion of private and shared CNA alterations across the different models (**D**). Boxplot showing the proportion of shared events in recurring bladder tumor samples (**E**)

Analysis of the SNP array data revealed that three models (Uro.1, GU.2, and BaSq.4) were diploid, and the remaining four were likely polyploid (GU.1, BaSq.1–3). All models exhibited chromosomal aberrations (Fig. 5A), with total copy number alterations (CNAs) ranging from 14 in Uro.1 to 50 in BaSq.3. Most CNAs were present in all samples over time and across locations, including complex rearrangements (Fig. 5B). We called shared and private CNAs for each model to test for ongoing chromosomal instability and to identify clonal and subclonal alterations during xenograft propagation in different microenvironments (Fig. 6A). Most models showed a few large segmental CNAs during passaging, with a median of 7 events per model (range: 2 in GU.2 to 16 in BaSq.2). While most CNAs were consistent across samples and locations, some alterations were absent in intermediate samples but reemerged in later samples. This pattern likely reflects regional heterogeneity in the sampled tumor areas rather than true biological evolution (e.g., BaSq.2, chr4 deletion in the SC lineage; Fig. 6A). Additionally, some of the shared CNAs (detected in at least two samples) were absent in the founding population/stem, suggesting that subclonal events were undetected in the primary samples. True adaptation to a specific microenvironment would be expected to drive lineage-specific longitudinal clonal expansion, yet only one CNA underwent clonal sweep across all samples: a chr16:1–33 Mb deletion in BaSq.1 (Fig. 6A). Further lineage-specific analyses of the GU.1, BaSq.2, and Uro.1 models revealed distinct patterns of clonal evolution with clonal sweeps across the subcutaneous, kidney, and fat pad lineages with less branching (median complexity <-0.45). BaSq.2 displayed the most clonal sweeps (five events) (Fig. 6A), whereas GU.1 was dominated by private aberrations leading to more branching and higher complexity scores (between − 0.35 and − 0.39), and Uro.1 showed parallel subclonal maintenance without significant clonal replacement (Fig. 6A and Supplementary Figs. 12–14). In BaSq.2, clonal replacement events (e.g., sample 3P4SC_7B, Fig. 6C) suggest the expansion of fitter subclones following mechanical bottlenecks during seeding (Fig. 6A-C). This was further supported by metastases and recurrences, where multiple acquired CNAs underwent clonal sweeps, indicating selection of fitter clones at metastatic sites (e.g., 3P6IC_3-SR vs. 3P6TV_2-Lg, 3P7SC_2B vs. 3P11TV_1, Supplementary Fig. 14). In contrast, metastases in GU.1 and Uro.1 showed minimal branching, with the clonal compositions largely unchanged (Supplementary Figs. 12 and 13). Overall, metastases and recurrent tumors were characterized by subclonal branching rather than a widespread clonal replacement (median complexity − 0.37), suggesting model-specific dynamics of chromosomal instability and clonal evolution.

Approximately 15% of CNAs were private, appearing in only one sample and typically in a subclonal context (Fig. 6A). New events did not cluster on specific chromosomes or occur in specific lineages, ruling out parallel evolution or lineage-specific fixation as driving factors. To compare chromosomal stability within models to that of recurrent bladder tumors, we analyzed ASCAT-estimated CNA events in a published patient cohort [43]. Asynchronous tumor samples shared an average of 25% of CNA events (16 patients, 1.8–75%), whereas synchronous samples exhibited a higher average of 31% shared events (6 patients, 10.2–78%) (Fig. 6E). In contrast, PDX samples from distinct anatomic sites demonstrated a significantly higher degree of similarity, with over 80% of CNA events shared across time points and anatomical locations (Fig. 6D).

## Discussion

In this study, we show that the phenotype and genotype of bladder tumors remain remarkably stable during growth in multiple organ-specific environments, including S.C. fat pad and kidney parenchyma as well as in metastatic sites. Molecular subtype classifications, based on gene and protein expression profiles, remained stable over time, with key tumor suppressor genes and oncogenes (RB1, TP53, CDKN2A/p16, FGFR3, and PIK3CA) showing no evidence of emerging intratumor heterogeneity (ITH). A previous study on subcutaneous PDXs reported intermediate subtype scores and variable GATA3 and KRT5/6 staining, described as subtype ITH in some cases [44]. In contrast, our analysis focused on cases with distinct subtype profiles, without baseline subtype ITH, and followed the models longitudinally without detecting new subtype heterogeneity. The TMB and mutation profiles remained stable across different environments, suggesting minimal genomic adaptation during tumor progression and metastasis. While private mutations were rare and likely associated with regional heterogeneity or technical variability, one *de novo* mutation in BCL7A in Gu.2 was detected in the FP samples. This suggests potential selection in that particular environment, although additional experimental validation is needed.

Copy number alterations (CNA) were similarly stable over time and across different environments, with most aberrations persisting throughout passaging, although some heterogeneity was also observed. Selection of preexisting minor clones and the accumulation of new CNAs during PDX passaging have been reported previously, particularly during the initial establishment passages [45]. Consistent with our observations, CNA changes for bladder cancer PDXs averaged ~ 6%, one of the lowest rates among all tumor types [46]. Thus, CNA differences between PDXs and primary tumors are similar to those observed across multiregional primary tumor samples [47], and tumor stability during PDX passaging has been confirmed in multiple studies. In our study, we sought to isolate the specific effects of different microenvironments and metastases on tumor evolution. Compared with recurrent patient-matched tumors, PDX tumors presented significantly higher CNA similarity across primary locations and metastases [43], emphasizing that no major genomic evolution occurs at this stage of tumor development regardless of the organ-specific environment.

For the first time, we report an in-depth characterization of spontaneous and induced metastasis in PDX models derived from human bladder tumors. The development of spontaneous metastasis after subcutaneous inoculation of patient-derived bladder tumors is rare and has, to our knowledge, been reported only recently in the MIBC BL0293 PDX model developed by Jackson Laboratory and the University of California-Davis [25, 48, 49]. In our study, extended mouse survival following primary tumor resection allowed the development of larger lung metastases. However, as only one H&E-stained lung section per mouse was analyzed, the presence of metastatic foci in the lungs or other organs may be underestimated. Tail vein injection of cancer cells primarily induces lung, liver, pleural, and mediastinal metastases [50, 51]. In contrast, intracardiac injection enables arterial dissemination, leading to metastases in bone, brain, liver, adrenal glands, kidneys, and ovaries [52, 53]. In our study, in addition to lung metastases, we observed more visceral metastases after intracardiac injection, particularly to the liver and suprarenal glands. In the Uro.1 model, visceral metastases were found following both tail vein and intracardiac injection. The brain and bones were not specifically investigated because of the absence of cell labeling; thus, their presence cannot be excluded. For the GU.2 model, which was derived from a patient with metastatic disease, it would have been valuable to analyse paired primary and metastatic tumor samples; however, these were not available to us. Taken together, spontaneous micrometastases to the lungs were detected in five models, while induced metastases spread more broadly, also to visceral organs. These findings underscore the value of these models for future studies on subtype-dependent metastatic mechanisms and organotropism.

Diverging genomic evolution between primary and metastatic BC has been described previously [54]. Despite the high number of CNAs in bladder cancer few of these seem to result in a phenotypic change or fitness gain that drives tumor growth during progression. Consistent with this, most of the CNAs were shared, and there were occasional subclonal variations, but clonal sweeps were rare. To separate subclonal variability due to sampling from true subclonal events, we considered the subclones that were longitudinally present in a lineage to be biologically meaningful. The BaSq.2 model was the most dynamic, with fitter subclones expanding during propagation and metastasis. Notably, while metastases had more subclonal branching than other locations did, only a few CNAs underwent true clonal selection, further supporting the notion that bladder cancer progression is driven primarily by preexisting features rather than late genomic evolution.

While bulk transcriptomic and immunohistochemical analyses did not reveal major subtype plasticity, we identified microenvironment-driven transcriptional adaptations, particularly in kidney capsule (KD) tumors and metastases. These changes, although not altering core subtype features, involved genes associated with hypoxia, inflammation, extracellular matrix remodeling, and adhesion, reflecting the dynamic interactions between tumor cells and their surroundings. Notably, many of the shared differentially expressed genes (DEGs) in metastases were also altered in KD tumors, suggesting that these are not metastasis-specific adaptations. The upregulation of *ZEB2* in multiple models and the consistent increase in *CYP24A1* expression across all metastases highlight potential mechanisms driving invasion and metastatic progression. Notably, metastatic samples from all molecular subtypes presented elevated *CYP24A1* expression, a key negative regulator of active vitamin D (calcitriol). Given that calcitriol inhibits COX-2 (*PTGS2*) and reduces PGE2 production [55], the concurrent upregulation of *PTGS2* in metastases suggests dysregulation of this pathway. Previous transcriptomic analyses of serially passaged metastatic BC cell lines in mice also identified *PTGS2* among the top five genes with over twofold upregulation in the highly metastatic KK-47HM4 subline [56], indicating a possible dependence on COX-2 activation for metastatic dissemination of certain BC phenotypes. Furthermore, a recent study demonstrated prolonged progression-free survival following aspirin treatment in PI3K-activated colorectal cancer [57]. Notably, both BC models with elevated COX-2 expression in metastases had PIK3CA driver mutations, suggesting a possible therapeutic benefit of COX-2 inhibition in PI3K-altered urothelial carcinoma. Most of these adaptations appear model- and context dependent, with no microenvironment-induced transcriptional changes shared by all models and subtypes. Future studies using spatial profiling or single-cell transcriptomics could also provide deeper insights into subtle cellular heterogeneity and microenvironmental influences that may not be captured by bulk analyses.

ITH can emerge from both stability and plasticity, driven by ongoing mutational processes, selective pressures, and epigenetic reprogramming. An appraisal of subtype ITH might have clinically important implications when treating metastatic disease, where therapeutic decisions, including targeted therapies, are often based on analyses of the primary tumor rather than the metastases [35]. At the multiomic level, bladder cancer is among the most heterogeneous tumor types [58]. Following the molecular classification, the extent of ITH and plasticity/stability during tumor progression and metastasis is only beginning to be elucidated, with different studies reaching contrasting conclusions regarding its existence and extent in bladder cancer [9, 10, 14, 15, 59, 60]. Implementing IHC-based subtyping in the pathology laboratory could serve as a critical bridge for integrating molecular stratification into routine clinical practice. A key advantage of in situ molecular assays is their ability to visualize intratumor heterogeneity across entire tumor sections. In our study, consistency in growth patterns, histological features, and genomic profiles across different anatomical sites and metastases indicates a strong intrinsic tumor identity that is not easily reprogrammed by the microenvironment.

Despite our comprehensive multiomic approach, the use of PDX models has several limitations, such as the absence of an immune system and the potential mismatch in ligand-receptor interactions between human tumor cells and the murine host. While our use of NSG mice allowed us to isolate the influence of local tissue microenvironments on tumor phenotype, the absence of adaptive immunity limits the ability to fully model immune-mediated selection pressures, which are known to vary across organs and play an important role in shaping tumor evolution. Similarly, our metastatic models reflect only hematogenous dissemination and do not recapitulate the lymphatic spread commonly observed in BC patients. Thus, our results do not fully reflect tumor evolution in a clinical setting, such as tumor adaptations in response to cytotoxic innate and adaptive immune cells. Moreover, our experimental system does not replicate the treatment-specific selective pressures that are likely to drive tumor evolution in patients undergoing successive therapeutic regimens. Finally, our results reflect tumor adaptation and evolution over a 3- to 7-months period in already established MIBCs, and more gradual changes occurring over longer timescales are unlikely to be detected by this model.

In summary, we extensively tested the potential impact of different microenvironments on BC tumor adaptation. Despite the inherently high tumor mutational burden and genomic complexity characteristic of bladder cancer [61, 62], our findings revealed no evidence of ongoing genomic instability during tumor progression and metastatic dissemination. This finding supports the model of punctuated evolution, as observed in other tumor types [63–65]. Bladder tumors have the capacity to grow and adapt to diverse microenvironments while maintaining genotype, clonal composition, and phenotypic state, highlighting remarkable stability during progression and metastasis.

## Electronic supplementary material

Below is the link to the electronic supplementary material.

**Supplementary Material 1**: The file with supplementary material includes Supplementary methods, Supplementary Tables 1–3, and Supplementary Figs. 1–13.

## Data Availability

Data is provided within the manuscript or supplementary information files.

## Abbreviations

BaSq: Basal/Squamous-like
BC: Bladder cancer
CNA: Copy number alterations
ECM: Extracellular matrix
EMT: Epithelial-mesenchymal transition
FP: Fat pad
IHC: Immunohistochemistry
ITH: Intratumor heterogeneity
GU: Genomically Unstable
NMIBC: Non-muscle invasive bladder cancer
MIBC: Muscle invasive bladder cancer
MCF: Mutated cell fractions
Mes-like: Mesenchymal-like
KD: Under the kidney capsule
PDX: Patient-derived xenografts
PBS: Phosphate-buffered saline
Sc/NE: Small cell Neuroendocrine-like
SC: Subcutaneous
TMA: Tissue microarray
TME: Tumor microenvironment
TMB: Tumor mutation burden
Uro: Urothelial-like

## References

[1] Ferlay J, Ervik M, Lam F, Laversanne M, Colombet M, Mery L et al. accessed December 23,. Global Cancer Observatory: Cancer Today. Lyon, France: International Agency for Research on Cancer. Global Cancer Observatory: Cancer Today Lyon, France: International Agency for Research on Cancer 2024. https://gco.iarc.who.int/today (2024).

[2] Alfred Witjes J, Max Bruins H, Carrión A, Cathomas R, Compérat E, Efstathiou JA, et al. European association of urology guidelines on Muscle-invasive and metastatic bladder cancer: summary of the 2023 guidelines. Eur Urol. 2024;85:17–31. 10.1016/j.eururo.2023.08.016. 37858453 10.1016/j.eururo.2023.08.016

[3] Galsky MD, Witjes JA, Gschwend JE, Milowsky MI, Schenker M, Valderrama BP, et al. Adjuvant nivolumab in High-Risk Muscle-Invasive urothelial carcinoma: expanded efficacy from checkmate 274. J Clin Oncol. 2025;43:15–21. 10.1200/JCO.24.00340. 39393026 10.1200/JCO.24.00340 PMC11687940

[4] Kamoun A, de Reyniès A, Allory Y, Sjödahl G, Robertson AG, Seiler R, et al. A consensus molecular classification of Muscle-invasive bladder Cancer. Eur Urol. 2020;77:420–33. 10.1016/j.eururo.2019.09.006. 31563503 10.1016/j.eururo.2019.09.006 PMC7690647

[5] Damrauer JS, Hoadley KA, Chism DD, Fan C, Tignanelli CJ, Wobker SE, et al. Intrinsic subtypes of high-grade bladder cancer reflect the hallmarks of breast cancer biology. Proc Natl Acad Sci U S A. 2014;111:3110–5. 10.1073/PNAS.1318376111. 24520177 10.1073/pnas.1318376111 PMC3939870

[6] Robertson AG, Kim J, Al-Ahmadie H, Bellmunt J, Guo G, Cherniack AD, et al. Comprehensive molecular characterization of Muscle-Invasive bladder Cancer. Cell. 2017;171:540–e55625. 10.1016/j.cell.2017.09.007. 28988769 10.1016/j.cell.2017.09.007 PMC5687509

[7] Choi W, Czerniak B, Ochoa A, Su X, Siefker-Radtke A, Dinney C, et al. Intrinsic basal and luminal subtypes of muscle-invasive bladder cancer. Nat Rev Urol. 2014;11:400–10. 10.1038/nrurol.2014.129. 24960601 10.1038/nrurol.2014.129

[8] Cotillas EA, Bernardo C, Veerla S, Liedberg F, Sjödahl G, Eriksson P. A versatile and upgraded version of the LundTax classification algorithm applied to independent cohorts. J Mol Diagn. 2024;26:1081–101. 10.1016/j.jmoldx.2024.08.005. 39326668 10.1016/j.jmoldx.2024.08.005

[9] Jakobsson L, Chebil G, Marzouka N-A-D, Liedberg F, Sjödahl G. Low frequency of intratumor heterogeneity in bladder Cancer tissue microarrays. Bladder Cancer. 2018;4:327–37. 10.3233/BLC-180176. 30112444 10.3233/BLC-180176 PMC6087434

[10] Sjödahl G, Eriksson P, Patschan O, Marzouka N, Jakobsson L, Bernardo C, et al. Molecular changes during progression from nonmuscle invasive to advanced urothelial carcinoma. Int J Cancer. 2020;146:2636–47. 10.1002/ijc.32737. 31609466 10.1002/ijc.32737 PMC7079000

[11] da Costa JB, Gibb EA, Nykopp TK, Mannas M, Wyatt AW, Black PC. Molecular tumor heterogeneity in muscle invasive bladder cancer: biomarkers, subtypes, and implications for therapy. Urologic Oncology: Seminars Original Investigations. 2022;40:287–94. 10.1016/j.urolonc.2018.11.015. 30528886 10.1016/j.urolonc.2018.11.015

[12] Schallenberg S, Dragomir MP, Anders P, Ebner B, Volz Y, Eismann L, et al. Intratumoral heterogeneity of molecular subtypes in Muscle-invasive bladder Cancer-An extensive multiregional immunohistochemical analysis. Eur Urol Focus. 2023;9:788–98. 10.1016/J.EUF.2023.03.012. 37076398 10.1016/j.euf.2023.03.012

[13] Olah C, Shmorhun O, Klamminger GG, Rawitzer J, Sichward L, Hadaschik B, et al. Immunohistochemistry-based molecular subtypes of urothelial carcinoma derive different survival benefit from platinum chemotherapy. J Pathol Clin Res. 2025;11:e70017. 10.1002/2056-4538.70017. 39817402 10.1002/2056-4538.70017 PMC11736421

[14] Sfakianos JP, Daza J, Hu Y, Anastos H, Bryant G, Bareja R, et al. Epithelial plasticity can generate multi-lineage phenotypes in human and murine bladder cancers. Nat Commun. 2020;11:2540. 10.1038/s41467-020-16162-3. 32439865 10.1038/s41467-020-16162-3 PMC7242345

[15] Warrick JI, Hu W, Yamashita H, Walter V, Shuman L, Craig JM, et al. FOXA1 repression drives lineage plasticity and immune heterogeneity in bladder cancers with squamous differentiation. Nat Commun 2022. 2022;13:1. 10.1038/s41467-022-34251-3. 10.1038/s41467-022-34251-3 PMC9630410 36323682

[16] Yang G, Bondaruk J, Cogdell D, Wang Z, Lee S, Lee JG et al. Urothelial-to-Neural Plasticity Drives Progression to Small Cell Bladder Cancer. IScience. 2020;23:101201. 10.1016/J.ISCI.2020.101201 10.1016/j.isci.2020.101201 PMC7286965 32521509

[17] Pérez-González A, Bévant K, Blanpain C. Cancer cell plasticity during tumor progression, metastasis and response to therapy. Nat Cancer. 2023;4:1063. 10.1038/S43018-023-00595-Y. 37537300 10.1038/s43018-023-00595-y PMC7615147

[18] Thomsen MBH, Nordentoft I, Lamy P, Høyer S, Vang S, Hedegaard J, et al. Spatial and Temporal clonal evolution during development of metastatic urothelial carcinoma. Mol Oncol. 2016;10:1450–60. 10.1016/j.molonc.2016.08.003. 27582092 10.1016/j.molonc.2016.08.003 PMC5423216

[19] Cox A, Klümper N, Stein J, Sikic D, Breyer J, Bolenz C, et al. Molecular urothelial tumor cell subtypes remain stable during metastatic evolution. Eur Urol. 2024;85:328–32. 10.1016/J.EURURO.2023.03.020. 37031005 10.1016/j.eururo.2023.03.020

[20] Wullweber A, Strick R, Lange F, Sikic D, Taubert H, Wach S, et al. Bladder tumor subtype commitment occurs in carcinoma In situ driven by key signaling pathways including ECM remodeling. Cancer Res. 2021;81:1552–66. 10.1158/0008-5472.CAN-20-2336. 33472889 10.1158/0008-5472.CAN-20-2336

[21] Egeblad M, Nakasone ES, Werb Z. Tumors as organs: complex tissues that interface with the entire organism. Dev Cell. 2010;18:884–901. 10.1016/j.devcel.2010.05.012. 20627072 10.1016/j.devcel.2010.05.012 PMC2905377

[22] Lee Y-C, Lam H-M, Rosser C, Theodorescu D, Parks WC, Chan KS. The dynamic roles of the bladder tumour microenvironment. Nat Rev Urol. 2022;19:515–33. 10.1038/s41585-022-00608-y. 35764795 10.1038/s41585-022-00608-y PMC10112172

[23] Sflomos G, Dormoy V, Metsalu T, Jeitziner R, Battista L, Scabia V, et al. A preclinical model for ERα-Positive breast Cancer points to the epithelial microenvironment as determinant of luminal phenotype and hormone response. Cancer Cell. 2016;29:407–22. 10.1016/j.ccell.2016.02.002. 26947176 10.1016/j.ccell.2016.02.002

[24] Wang Y, Revelo MP, Sudilovsky D, Cao M, Chen WG, Goetz L, et al. Development and characterization of efficient xenograft models for benign and malignant human prostate tissue. Prostate. 2005;64:149–59. 10.1002/pros.20225. 15678503 10.1002/pros.20225

[25] Jäger W, Xue H, Hayashi T, Janssen C, Awrey S, Wyatt AW, et al. Patient-derived bladder cancer xenografts in the preclinical development of novel targeted therapies. Oncotarget. 2015;6:21522. 10.18632/oncotarget.3974. 26041878 10.18632/oncotarget.3974 PMC4673283

[26] Abu Quora HA, Zahra MH, El-Ghlban S, Nair N, Afify SM, Hassan G, et al. Microenvironment of mammary fat pads affected the characteristics of the tumors derived from the induced cancer stem cells. Am J Cancer Res. 2021;11:3475–95. http://www.ncbi.nlm.nih.gov/pubmed/34354856 34354856 PMC8332865

[27] Neville MC, Medina D, Monks J, Hovey RC. The mammary fat pad. J Mammary Gland Biol Neoplasia. 1998;3:109–16. 10.1023/A:1018786604818. 10819521 10.1023/a:1018786604818

[28] Elliott BE, Tam S-P, Dexter D, Chen ZQ. Capacity of adipose tissue to promote growth and metastasis of a murine mammary carcinoma: effect of Estrogen and progesterone. Int J Cancer. 1992;51:416–24. 10.1002/IJC.2910510314. 1317363 10.1002/ijc.2910510314

[29] Nieman KM, Kenny HA, Penicka CV, Ladanyi A, Buell-Gutbrod R, Zillhardt MR, et al. Adipocytes promote ovarian cancer metastasis and provide energy for rapid tumor growth. Nat Med 2011. 2011;17:11. 10.1038/nm.2492. 10.1038/nm.2492 PMC4157349 22037646

[30] Wang YY, Attané C, Milhas D, Dirat B, Dauvillier S, Guerard A, et al. Mammary adipocytes stimulate breast cancer invasion through metabolic remodeling of tumor cells. JCI Insight. 2017;2:e87489. 10.1172/jci.insight.87489. 28239646 10.1172/jci.insight.87489 PMC5313068

[31] Zhu Q, Zhu Y, Hepler C, Zhang Q, Park J, Gliniak C, et al. Adipocyte mesenchymal transition contributes to mammary tumor progression. Cell Rep. 2022;40:111362. 10.1016/j.celrep.2022.111362. 36103820 10.1016/j.celrep.2022.111362 PMC9533474

[32] Sun HZ, Wu SF, Tu ZH. Blockage of IGF-1R signaling sensitizes urinary bladder cancer cells to mitomycin-mediated cytotoxicity. Cell Research. 2001 11:2 2001;11:107–15. 10.1038/sj.cr.7290075 10.1038/sj.cr.7290075 11453542

[33] Mukae Y, Miyata Y, Nakamura Y, Araki K, Otsubo A, Yuno T, et al. Pathological roles of c-Met in bladder cancer: association with cyclooxygenase-2, Heme oxygenase-1, vascular endothelial growth factor-A and programmed death ligand 1. Oncol Lett. 2020;20:135. 10.3892/OL.2020.11540. 32565941 10.3892/ol.2020.11540 PMC7285828

[34] Memon AA, Sorensen BS, Melgard P, Fokdal L, Thykjaer T, Nexo E. Expression of HER3, HER4 and their ligand heregulin-4 is associated with better survival in bladder cancer patients. Br J Cancer 2004;91:2034. 10.1038/SJ.BJC.6602251 10.1038/sj.bjc.6602251 PMC2409781 15583696

[35] Klümper N, Cox A, Sjödahl G, Roghmann F, Bolenz C, Hartmann A, et al. Pre-treatment metastatic biopsy: a step towards precision oncology for urothelial cancer. Nat Rev Urol. 2024. 10.1038/S41585-024-00951-2. 39472646 10.1038/s41585-024-00951-2

[36] Sjödahl G, Abrahamsson J, Holmsten K, Bernardo C, Chebil G, Eriksson P, et al. Different responses to neoadjuvant chemotherapy in urothelial carcinoma molecular subtypes. Eur Urol. 2022;81:523–32. 10.1016/j.eururo.2021.10.035. 34782206 10.1016/j.eururo.2021.10.035

[37] Conway T, Wazny J, Bromage A, Tymms M, Sooraj D, Williams ED, et al. Xenome—a tool for classifying reads from xenograft samples. Bioinformatics. 2012;28:i172–8. 10.1093/BIOINFORMATICS/BTS236. 22689758 10.1093/bioinformatics/bts236 PMC3371868

[38] Garcia M, Juhos S, Larsson M, Olason PI, Martin M, Eisfeldt J, et al. Sarek: A portable workflow for whole-genome sequencing analysis of germline and somatic variants. F1000Res. 2020;9:63. 10.12688/F1000RESEARCH.16665.2. 32269765 10.12688/f1000research.16665.1 PMC7111497

[39] Rasmussen M, Sundström M, Göransson Kultima H, Botling J, Micke P, Birgisson H, et al. Allele-specific copy number analysis of tumor samples with aneuploidy and tumor heterogeneity. Genome Biol. 2011;12:R108. 10.1186/gb-2011-12-10-r108. 22023820 10.1186/gb-2011-12-10-r108 PMC3333778

[40] Andersson N, Chattopadhyay S, Valind A, Karlsson J, Gisselsson D. DEVOLUTION-A method for phylogenetic reconstruction of aneuploid cancers based on multiregional genotyping data. Commun Biol. 2021;4. 10.1038/S42003-021-02637-6. 10.1038/s42003-021-02637-6 PMC8452746 34545199

[41] Schliep KP. Phangorn: phylogenetic analysis in R. Bioinformatics. 2011;27:592–3. 10.1093/BIOINFORMATICS/BTQ706. 21169378 10.1093/bioinformatics/btq706 PMC3035803

[42] Bernardo C, Eriksson P, Marzouka N, Liedberg F, Sjödahl G, Höglund M. Molecular pathology of the luminal class of urothelial tumors. J Pathol. 2019;249:308–18. 10.1002/path.5318. 31232464 10.1002/path.5318 PMC6851980

[43] Marzouka N-A-D, Lindgren D, Eriksson P, Sjödahl G, Bernardo C, Liedberg F, et al. Recurring urothelial carcinomas show genomic rearrangements incompatible with a direct relationship. Sci Rep. 2020;10:19539. 10.1038/s41598-020-75854-4. 33177554 10.1038/s41598-020-75854-4 PMC7658206

[44] Lang H, Béraud C, Cabel L, Fontugne J, Lassalle M, Krucker C, et al. Integrated molecular and Pharmacological characterization of patient-derived xenografts from bladder and ureteral cancers identifies new potential therapies. Front Oncol. 2022;12:930731. 10.3389/fonc.2022.930731 36033544 10.3389/fonc.2022.930731 PMC9405192

[45] Ben-David U, Ha G, Tseng Y-Y, Greenwald NF, Oh C, Shih J, et al. Patient-derived xenografts undergo mouse-specific tumor evolution. Nat Genet. 2017;49:1567–75. 10.1038/ng.3967. 28991255 10.1038/ng.3967 PMC5659952

[46] Hoge ACH, Getz M, Zimmer A, Ko M, Raz L, Beroukhim R, et al. DNA-based copy number analysis confirms genomic evolution of PDX models. NPJ Precis Oncol. 2022;6:30. 10.1038/s41698-022-00268-6. 35484194 10.1038/s41698-022-00268-6 PMC9050710

[47] Woo XY, Giordano J, Srivastava A, Zhao Z-M, Lloyd MW, de Bruijn R, et al. Conservation of copy number profiles during engraftment and passaging of patient-derived cancer xenografts. Nat Genet. 2021;53:86–99. 10.1038/s41588-020-00750-6. 33414553 10.1038/s41588-020-00750-6 PMC7808565

[48] Bernardo C, Costa C, Sousa N, Amado F, Santos L. Patient-derived bladder cancer xenografts: a systematic review. Translational Res. 2015;166:324–31. 10.1016/J.TRSL.2015.02.001. 10.1016/j.trsl.2015.02.001 25742701

[49] Tatum JL, Kalen JD, Jacobs PM, Ileva LV, Riffle LA, Hollingshead MG, et al. A spontaneously metastatic model of bladder cancer: imaging characterization. J Transl Med. 2019;17:1–10. 10.1186/S12967-019-02177-y 31878948 10.1186/s12967-019-02177-y PMC6931243

[50] Wong CW, Lee A, Shientag L, Yu J, Dong Y, Kao G et al. Apoptosis: an early event in metastatic inefficiency. Cancer Res 2001;61. http://www.ncbi.nlm.nih.gov/pubmed/11196183 11196183

[51] Kokabi F, Khosravi A, Jazi MS, Asadi J. A reliable mouse model of liver and lung metastasis by injecting esophageal cancer stem cells (CSCs) through tail-vein injection. Mol Biol Rep. 2023;50:3401–11. 10.1007/S11033-023-08294-8 36753017 10.1007/s11033-023-08294-8

[52] Khanna C, Hunter K. Modeling metastasis in vivo. Carcinogenesis. 2005;26:513–23. 10.1093/CARCIN/BGH261. 15358632 10.1093/carcin/bgh261

[53] Lu X, Kang Y. Organotropism of breast cancer metastasis. J Mammary Gland Biol Neoplasia. 2007;12:153–62. 10.1007/S10911-007-9047-3 17566854 10.1007/s10911-007-9047-3

[54] Faltas BM, Prandi D, Tagawa ST, Molina AM, Nanus DM, Sternberg C, et al. Clonal evolution of chemotherapy-resistant urothelial carcinoma. Nat Genet. 2016;48:1490–9. 10.1038/ng.3692. 27749842 10.1038/ng.3692 PMC5549141

[55] Wang Q, He Y, Shen Y, Zhang Q, Chen D, Zuo C, et al. Vitamin D inhibits COX-2 expression and inflammatory response by targeting thioesterase superfamily member 4. J Biol Chem. 2014;289:11681–94. 10.1074/jbc.M113.517581. 24619416 10.1074/jbc.M113.517581 PMC4002078

[56] Sugiyama N, Yoneyama MS, Hatakeyama S, Yamamoto H, Okamoto A, Koie T, et al. In vivo selection of high-metastatic subline of bladder cancer cell and its characterization. Oncol Res. 2013;20:289–95. 10.3727/096504013X13639794277644. 23879169 10.3727/096504013x13639794277644

[57] Liao X, Lochhead P, Nishihara R, Morikawa T, Kuchiba A, Yamauchi M, et al. Aspirin use, tumor PIK3CA mutation, and Colorectal-Cancer survival. N Engl J Med. 2012;367:1596–606. 10.1056/NEJMOA1207756. 23094721 10.1056/NEJMoa1207756 PMC3532946

[58] Hoadley KA, Yau C, Hinoue T, Wolf DM, Lazar AJ, Drill E, et al. Cell-of-Origin patterns dominate the molecular classification of 10,000 tumors from 33 types of Cancer. Cell. 2018;173:291–e3046. 10.1016/J.CELL.2018.03.022. 29625048 10.1016/j.cell.2018.03.022 PMC5957518

[59] Fontugne J, Wong J, Cabel L, Neyret-Kahn H, Karboul N, Maillé P, et al. Progression-associated molecular changes in basal/squamous and sarcomatoid bladder carcinogenesis. J Pathol. 2023;259:455–67. 10.1002/PATH.6060. 36695554 10.1002/path.6060

[60] Lindskrog SV, Schmøkel SS, Nordentoft I, Lamy P, Knudsen M, Prip F, et al. Single-nucleus and spatially resolved intratumor subtype heterogeneity in bladder Cancer. Eur Urol Open Sci. 2023;51:78–88. 10.1016/j.euros.2023.03.006. 37187723 10.1016/j.euros.2023.03.006 PMC10175738

[61] Chalmers ZR, Connelly CF, Fabrizio D, Gay L, Ali SM, Ennis R, et al. Analysis of 100,000 human cancer genomes reveals the landscape of tumor mutational burden. Genome Med. 2017;9:34. 10.1186/s13073-017-0424-2. 28420421 10.1186/s13073-017-0424-2 PMC5395719

[62] Meeks JJ, Al-Ahmadie H, Faltas BM, Taylor JA, Flaig TW, DeGraff DJ, et al. Genomic heterogeneity in bladder cancer: challenges and possible solutions to improve outcomes. Nat Rev Urol. 2020;17:259–70. 10.1038/S41585-020-0304-1. 32235944 10.1038/s41585-020-0304-1 PMC7968350

[63] Sottoriva A, Kang H, Ma Z, Graham TA, Salomon MP, Zhao J, et al. A big Bang model of human colorectal tumor growth. Nat Genet. 2015;47. 10.1038/ng.3214. 10.1038/ng.3214 PMC4575589 25665006

[64] Kang H, Salomon MP, Sottoriva A, Zhao J, Toy M, Press MF, et al. Many private mutations originate from the first few divisions of a human colorectal adenoma. J Pathol. 2015;237. 10.1002/path.4581. 10.1002/path.4581 PMC4607608 26119426

[65] Stephens PJ, Greenman CD, Fu B, Yang F, Bignell GR, Mudie LJ et al. Massive genomic rearrangement acquired in a single catastrophic event during cancer development. Cell 2011;144. 10.1016/j.cell.2010.11.055 10.1016/j.cell.2010.11.055 PMC3065307 21215367

