# Supplementary material for: Bladder cancer subtypes exhibit limited plasticity across different microenvironments and in metastases
Source: Exp Hematol Oncol. 2025 Jul 2;14:91. doi: 10.1186/s40164-025-00682-z (PMC12225047; doi:10.1186/s40164-025-00682-z)
Supplement: Supplementary file 1 — Supplementary Material 1: The file with supplementary material includes Supplementary methods, Supplementary Tables 1–3, and Supplementary Figs. 1–13. [file 40164_2025_682_MOESM1_ESM.docx]

**Bladder cancer subtypes exhibit limited plasticity across different microenvironments and in metastases**

Carina Bernardo^1*^, Subhayan Chattopadhyay^2^, Natalie Andersson^2,3^, Pontus Eriksson^1^, Benjamin Medle^1^, Lena Tran^1^, Nour al dain Marzouka^4^, Adam Mattsson^1^, Aymeric Zadoroznyj^1^, Malin Larsson^5^, Fredrik Liedberg^6^, Mattias Höglund^1^, Gottfrid Sjödahl^6^

### Supplementary Methods

#### Sample processing

Tissue samples were lysed with RTL Plus lysis buffer (Qiagen) containing Dithiothreitol (DTT) and disrupted with Qiagen TissueLyser for 8 minutes. After disruption, the lysed samples were centrifuged in QIAshredder columns, and the lysates stored at -80°C until further processing. DNA and RNA were isolated via the AllPrep DNA/RNA mini kit in a QIAcube with standard protocols. RNA quality was evaluated with an Agilent 2100 Bioanalyzer system.

#### RNA data analysis

FASTQ files were preprocessed and aligned via the nf-core/rnaseq pipeline (v. 3.9) using *nextflow* (v. 22.04.5). The GRCh38 reference genome and gene annotations release 108 were downloaded from Ensembl. Alignment and quantification were performed with *STAR* (v. 2.7.10a) and *RSEM* (v. 1.3.1) after adapter and quality trimming (*trimgalore*: 0.6.7) and removal of ribosomal RNA (*SortMeRNA*: 4.3.4). *SAMtools* (v. 1.15.1) was used to sort and index alignments, Picard (v. 2.27.4-*SNAPSHOT*) was used to mark duplicates and *Stringtie* (v. 2.2.1) was used for transcript assembly and quantification for quality analysis. Quality control was performed via *FastQC* (v. 0.11.9) and *MultiQC* (v. 1.13). Samples with fewer than 15 million mapped reads were excluded from the analysis. The absence of significant batch effects was confirmed before proceeding with the analysis.

RSEM merged gene counts were used to identify differentially expressed genes (DEGs) with the *DESeq2* R package (v. 1.38.3). DEGs between locations for each model were determined using the likelihood ratio test (chi-square test) for generalized linear models (GLMs), which compares two negative binomial models (a null model with no group effect and an alternative model with a group effect) fitted to count data. Bonferroni correction was applied to control for false discoveries (type I errors) during multiple testing. Pairwise comparisons to identify genes with significant differences in the mean expression levels between each group and the SC group were performed using the Wald test with an adjusted p-value < 0.01 and an absolute log2FoldChange > 1 as screening criteria.

#### Phylogenetic analysis

After segmentation of the SNP array data using TAPs we noticed that some large segment calls were a mixture of smaller segments with differing clonality statuses. To remedy this, we used the DNAcopy package to call segments according to both relative coverage and allele frequency separately. Interestingly, these generate micro segments that are typically unsuitable for quality control. We then employed a Bayesian merging algorithm where each segment is merged with the following segment if the posterior segment does not differ significantly (confidence band of 20%) from the prior estimate, and the process is iterated to cover each chromosomal arm unless a break point is detected. At the breakpoint, the process starts again. Estimates from both calls were merged to create a consensus segment call. The logR and allelic imbalance values for these segments were then aggregated from the TAPS estimates of small regions. Notably, some small segments (<10 kb) with high ploidy status were detected in some samples, possibly indicating amplicons. These were excluded from the analysis unless they appeared in more than one consecutively passaged sample and could thus be confidently called as to have been carried forward. Although these can provide information on subclonal diversification, they were often not covered with an adequate number of probes to be reliably called across all samples in a single lineage. In general, only segments with lengths greater than or equal to 0.1 Mb were included in downstream phylogenetic analyses. The script for these steps can be found in (<https://github.com/Subhayan18/Bernardo_et.al.git>).

The final clonal deconvolution for phylogenetic analysis was based on the following rules (Karlsson et al., 2024):

- Owing to a 10% error margin at clone size estimation with the SNP array, the final estimated sizes were rounded to the nearest 5%, assuming a +/- 5% spread.
- The sum of subclone sizes was allowed to reach an addition of 20% over the 100% limit owing to the 10% additive error margin, as two concomitant subclones may yield a 20% overestimate in clone size.
- The tumor cell content was multiplicatively normalized to 100% if the total clone size sum exceeded 100%.
- Copy number neutral events in a sample against a background of hyperdiploidy (ex. 2+1) was assumed to occur by losing an allele from the minor branch (2+0 instead of 1+1) if many of the other samples from the same experiment also displayed the same ploidy base.

Segment-specific aggregated logR values were obtained from SNP-array analyses as described previously. The standard expression for the proportion of the total number of alleles at the site of analysis and a normalized normal copy number is as follows:

$logR$ = {MCF*(CNa + CNb) + (1-MCF)*CNb}/CNp

The copy number of the aberrant segment is CNa, the copy number of the background cells (either the number of alleles in the parental clone or that of the *normal* cells) is CNb, and the ploidy level to which the array is adjusted is CNp. To estimate the MCF, one can use the following formula:

$\hat{MCF}$= {CNp * $logR$ – CNb} / CNa

This estimate can vary depending on the sequencing platform and may require rescaling accordingly. The MCF value can also be estimated from the mirrored B-allele frequency (mBAF) assuming that for a set of mutations, the B-allele frequencies are greater than or equal to those of the other alleles (A). Hence,

mBAF = Nb/(Na+Nb)

Here, Nb and Na indicate the number of B and A alleles, respectively. The total number of B alleles given with Nb in a sample at a specific location is the sum of the B-allele fractions in the tumor and that in the normal whose relative fractions can be assumed to add up to one. Hence,

Nb = Nb_Tumor_ + Nb_Normal_ = B_Tumor_ * MCF + (B_Tumor_ + B_Normal_) * (1-MCF) and,

Na = Na_Tumor_ + Na_Normal_ = A_Tumor_ * MCF + (A_Tumor_ + A_Normal_) * (1-MCF)

which can be simplified to:

$\hat{MCF}$ = (1 – 2 mBAF) / {mBAF * (CNa+CNb-2) – CNb + 1}

The mBAF values can be estimated from TAPS-generated estimates of allelic imbalance surmised according to the segment calls:

$\hat{mBAF}$ = 0.5 + Allelic Imbalance * 0.5

Most basic measures describing the appearance of a phylogenetic tree overlook the complexity of the evolutionary network. For example, when a linear evolutionary pattern is compared with a branching evolutionary pattern, the tree length and number of subclones of two trees may be identical, yet the complexity of the branching pattern may be markedly different. To capture these differences, we introduced an additional network measure (Petersson et al., 2022). A phylogenetic tree, being a monotonic network, structurally resembles a decision tree, which is often used to support decision-making by predicting different outcomes based on various actions. Both structures feature nodes and branches, ending in terminal nodes or leaves, and start from a common point, such as the most recent common ancestor in clonal evolution. We translated the phylogenies into a decision tree format by assigning each node and branching point to a generation, with complexity varying at order O(2n) from 0 to 2^(i-1)^, I being the generation and n being the nodes at the i^th^ generation. The number of terminal nodes in each generation contributes to the complexity at order O(n) with the complexity measure ($Y$) ranging between 0.5 (purely branched evolution) and 2.0 (purely linear evolution). We perform a nonlinear one-to-one transformation on this measure to realign the metric centering at 0 ($\hat{Y}$ between -1 (purely branched) and 1 (purely linear)).

$$\hat{Y}= \frac{2(Y-0.5)}{1.5}-1$$

### References

Karlsson, J., Yasui, H., Mañas, A., Andersson, N., Hansson, K., Aaltonen, K., Jansson, C., Durand, G., Ravi, N., Ferro, M., Yang, M., Chattopadhyay, S., Paulsson, K., Spierings, D., Foijer, F., Valind, A., Bexell, D., & Gisselsson, D. (2024). Early evolutionary branching across spatial domains predisposes to clonal replacement under chemotherapy in neuroblastoma. *Nature Communications*, *15*(1), 8992. https://doi.org/10.1038/s41467-024-53334-x

Petersson, A., Andersson, N., Hau, S. O., Eberhard, J., Karlsson, J., Chattopadhyay, S., Valind, A., Elebro, J., Nodin, B., Leandersson, K., Gisselsson, D., & Jirström, K. (2022). Branching Copy-Number Evolution and Parallel Immune Profiles across the Regional Tumor Space of Resected Pancreatic Cancer. *Molecular Cancer Research*, *20*(5), 749–761. https://doi.org/10.1158/1541-7786.MCR-21-0986/680161/AM/BRANCHING-COPY-NUMBER-EVOLUTION-AND-PARALLEL

### Supplementary tables

| Model | JAX_ID | Histology | Grade/Stage | Gender/Age | Treatment naive |
| --- | --- | --- | --- | --- | --- |
| Uro.1 | J000104256 | UBC | NA | M/79 | yes |
| Uro.2 | TM01002(BL0591F) | Papillary UBC | LG/pTis N0 | M/65 | yes |
| GU.1 | J000101121 | UBC | HG/pT2 | M/67 | no |
| GU.2 | TM00023(BL0429F) | Adenocarcinoma | HG/pT4N3M1 | F/60 | yes |
| BaSq.1 | J000100646 | UBC | HG/NA | M/72 | yes |
| BaSq.2 | TM00026(BL0479F) | UBC | HG/pT2b | F/78 | yes |
| BaSq.3 | J000099207 | UBC | HG/NA | M/76 | no |
| BaSq.4 | TM01029(BL0215F) | Papillary UBC | HG/MIBC | M/77 | yes |

**Supplementary Table 1.** Clinical characteristics of donor patients. Additional data and information for the PDX models is available here: <https://tumor.informatics.jax.org/mtbwi/pdxSearch.do>.

**Supplementary Table 2.** List of primary antibodies and specific clones used for immunohistochemistry analysis.

| Marker | Dilution | Clone | Provider |
| --- | --- | --- | --- |
| CCNB1 | 1:50 | Y106 | LifeSpan-BioSciences |
| CK5 | 1:25 | EP1601Y | Thermo Scientific |
| CK14 | 1:50 | LL002 | Leica Biosystems |
| RB1 | 1:500 | 4H1 | Cell Signaling |
| PPARG | 1:50 | C26H12 | Cell Signaling |
| GATA3 | 1:800 | L50-823 | CellMarque |
| FGFR3 | 1:50 | B-9 | Santa Cruz Biotech |
| EGFR | 1:50 | E30 | Agilent Technologies |
| CDH3 | 1:200 | 56-pcadherin | BD bBioScience |
| CCND1 | 1:50 | EP12 | Agilent Technologies |
| P63 | 1:100 | 4A4 | Agilent Technologies |
| E2F3 | 1:100 | 3E2F04 | LS Bio |
| CDKN2A(p16) | RTU | E6H4 | Ventana |
| CK20 | 1:50 | Ks20.8 | Agilent Technologies |
| UPK2 | 1:100 | BC21 | Histolab |
| ASMA | 1:100 | D49N | Cell Signaling |

RTU: Ready-to-Use

**Supplementary Table 3.** PDX models with detected spontaneous lung metastases and number of mice with metastases after intracardiac (IC) or tail vein (TV) injection and location of the metastatic lesions. The presence of lung micrometastases was assessed using a single H&E-stained lung section per animal. Variables such as tissue processing and staining and the proportion of mice that remained under observation after primary tumor excision (in the case of spontaneous mets) likely affected the metastases detection. Lg: Lung, Panc: Pancreas, SR: Supra Renal, Lv: Liver, Ov: Ovary.

| Model | Spontaneous metastases | | Induced metastases | | | |
| --- | --- | --- | --- | --- | --- | --- |
|  | **Presence** | **Proportion**  **(mets/total)** | **IC (mets/total)** | **TV (mets/total)** | **Location** | **Total** |
| Uro.1 | Yes | 19/30 (63%) | 2/8 (25%) | 3/5 (60%) | Lg, Panc, SR | 5/13 (38%) |
| GU.1 | Yes | 10/41(24%) | 3/8 (38%) | 1/6 (16%) | Lg, Lv, SR, OV | 4/14 (29%) |
| GU.2 | Yes | 6/44 (13%) | 1/7 (14%) | 0/6 (0%) | Lg, Lv | 1/13 (7%) |
| BaSq.1 | No | - | 0/4 (0%) | 0/3 (0%) | - | 0/7 (0%) |
| BaSq.2 | Yes | 18/54(33%) | 6/6 (100%) | 4/4 (100%) | Lg, SR | 10/10 (100%) |
| BaSq.3 | Yes | 1/40(2%) | 1/6 (16%) | 0/2 (0%) | Lg | 1/8 (12%) |
| BaSq.4 | No | - | 0/9 (0%) | 0/4 (0%) | - | 0/13 (0%) |

### Supplementary Figures


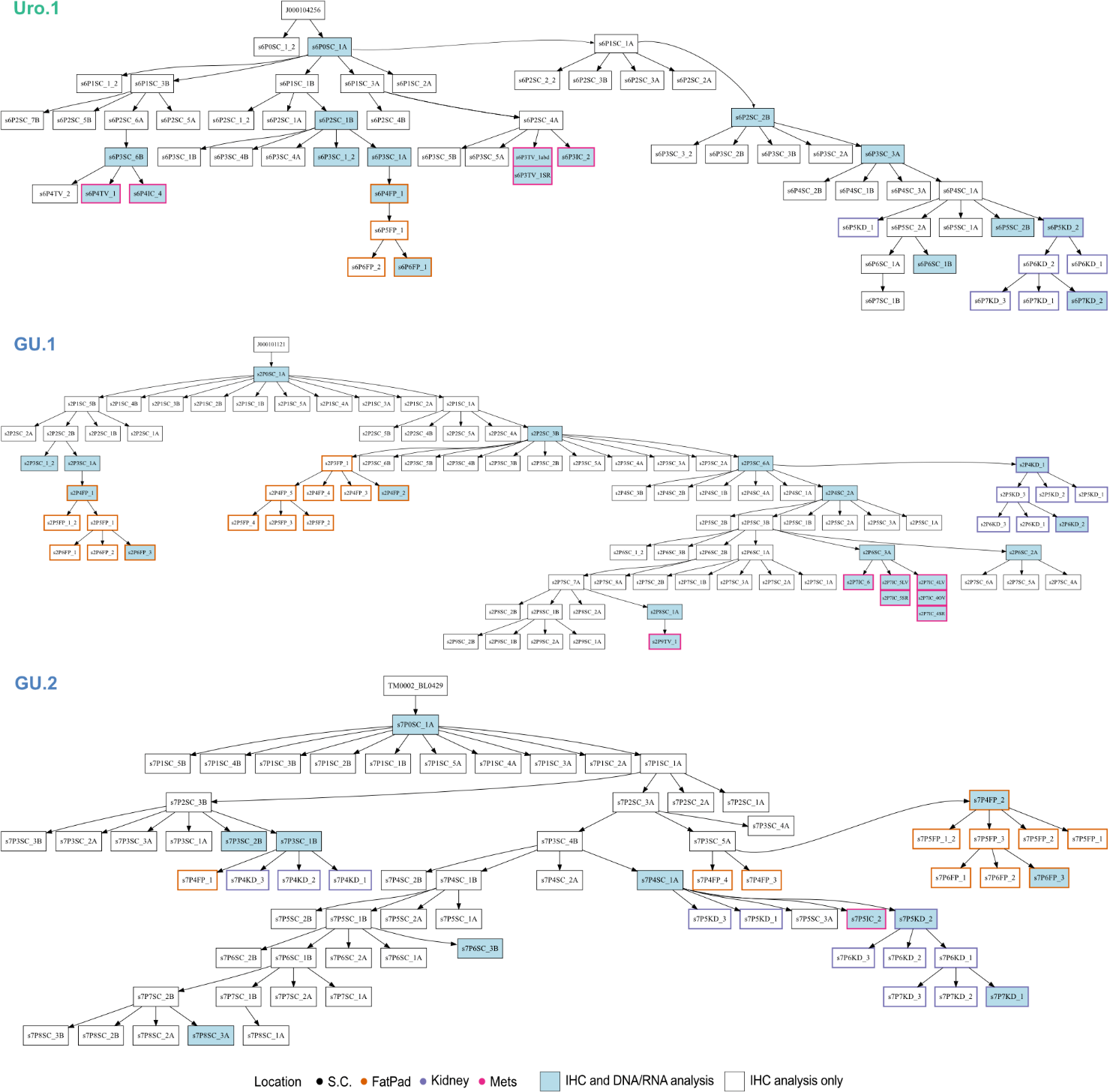


**Supplementary figure 1A.** Diagrams of the Uro and GU models showing the relationship between samples during propagation and growth under different environments. All successful tumors were processed for immunohistochemistry analysis. Blue shaded squares represent samples processed for DNA and RNA analysis. Color-coded boxes indicate the location of tumor implantation. All metastasis processed for DNA/RNA analysis were obtained from experimentally induced models. Multiple metastatic lesions obtained from the same mouse are indicated as stacked boxes.


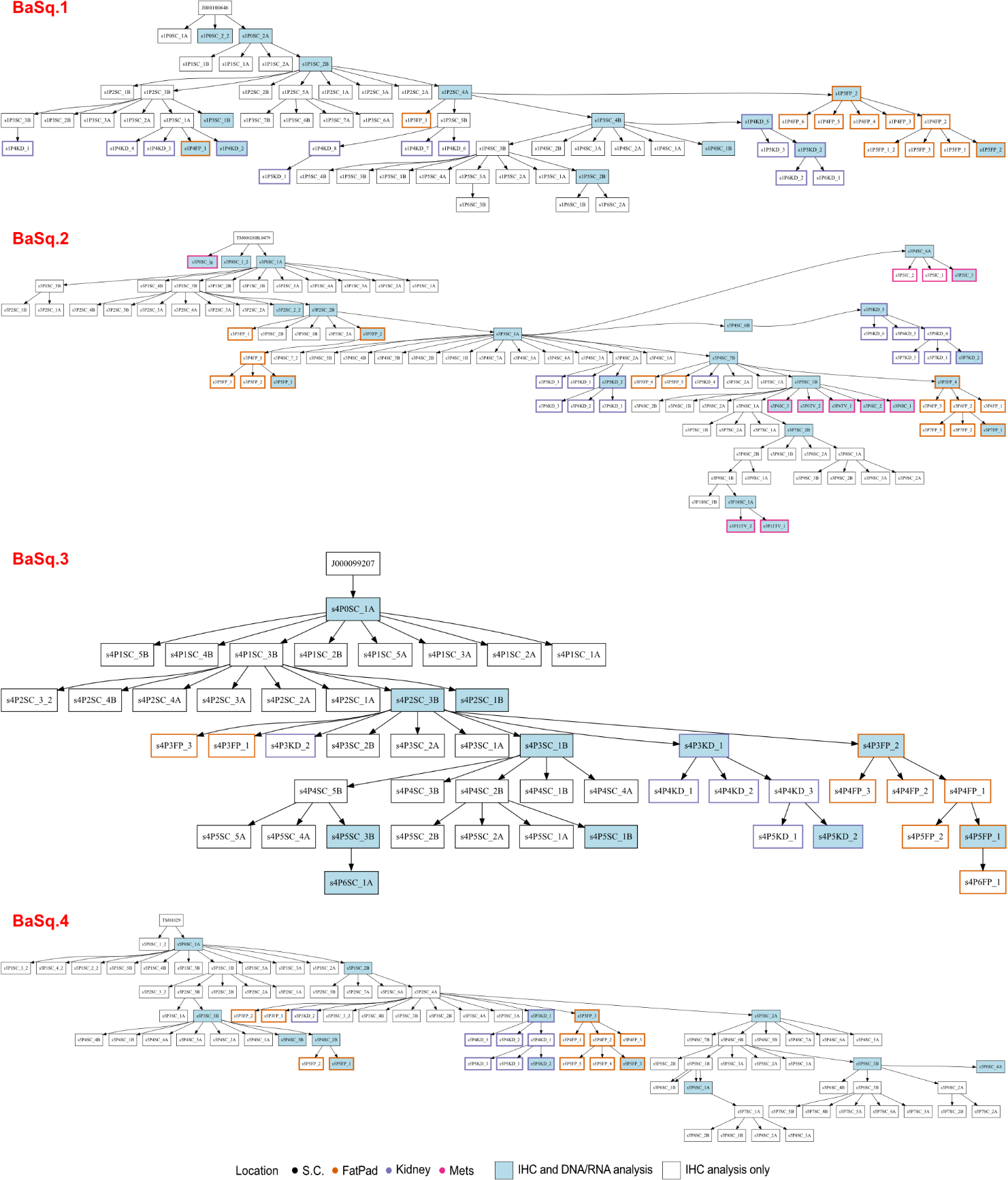


**Supplementary figure 1B.** Diagrams of the BaSq models showing the relationship between samples during propagation and growth under different environments. All successful tumors were processed for immunohistochemistry analysis. Blue shaded squares represent samples processed for DNA and RNA analysis. All metastasis processed for DNA/RNA analysis were obtained from experimentally induced models except for one spontaneous metastasis collected from the lungs in the BaSa.2 model. Color-coded boxes indicate the location of tumor implantation.


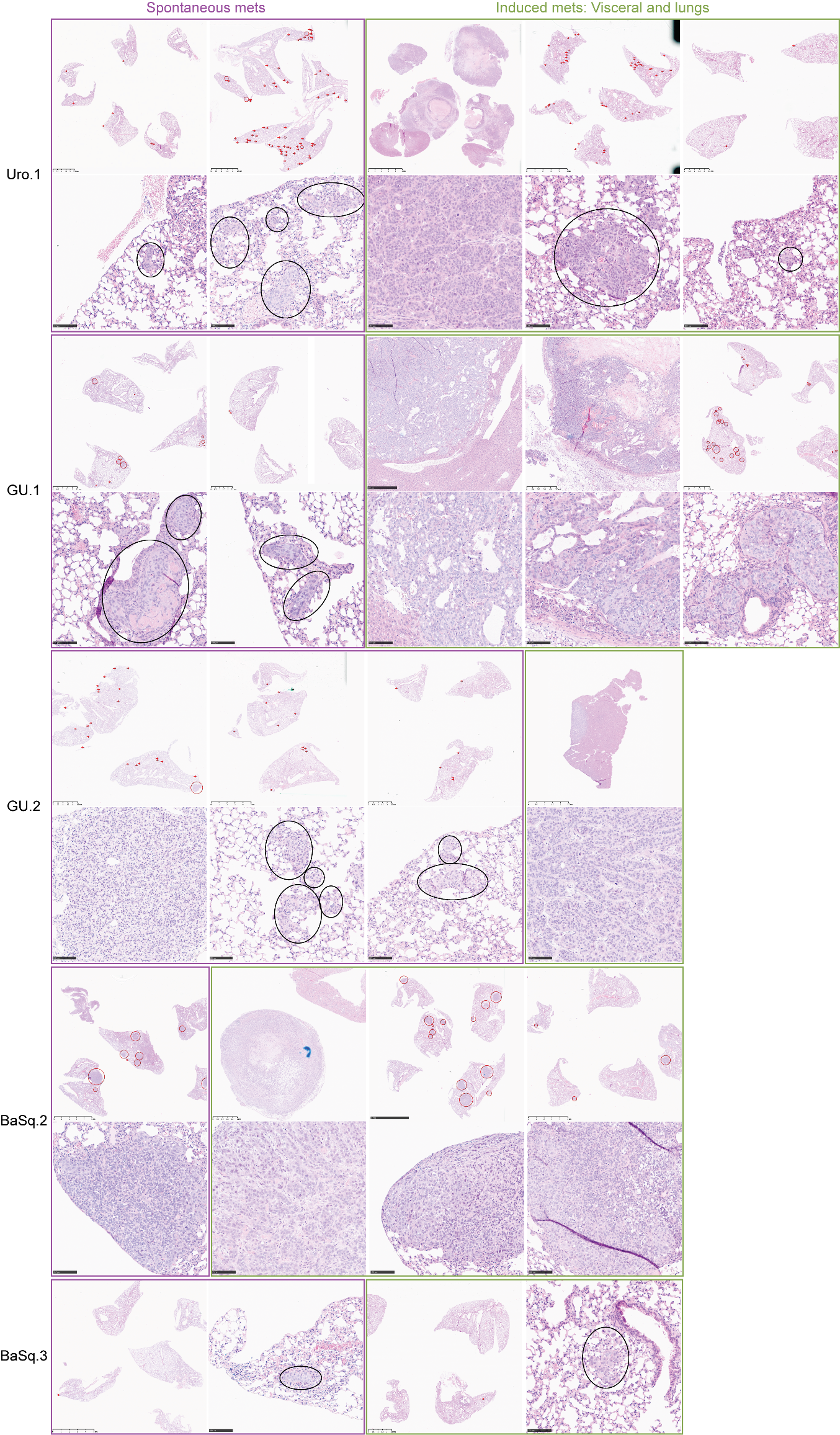


**Supplementary figure 2.** Representative images of the histology of spontaneous and induced metastases. For each model, the top panel shows an overview of the effected organ in the low magnification, were micrometastases are marked with red circles or arrows. The bottom panels show a closeup image of the image above with a 20X magnification where the scale bar indicates 100µm. Spontaneous metastases, arranged in purple rectangles, were exclusively observed in the lungs in mice after tumor growth in the subcutaneous (SC), fat pad (FP) or under the kidney capsule (KD) sites. Induced metastasis, arranged in green rectangles, developed after intracardiac (IC) or Tail vein (TV) injection of tumor cells. In these images we can appreciate differences between models that originate small/large metastatic foci, singular lesions and multiple lesions. In addition to lung metastases, images of visceral metastases are show for all induced models, except for BaSq.3 which did not generate visceral mets. DNA and RNA were extracted from macrometastases for downstream analysis.


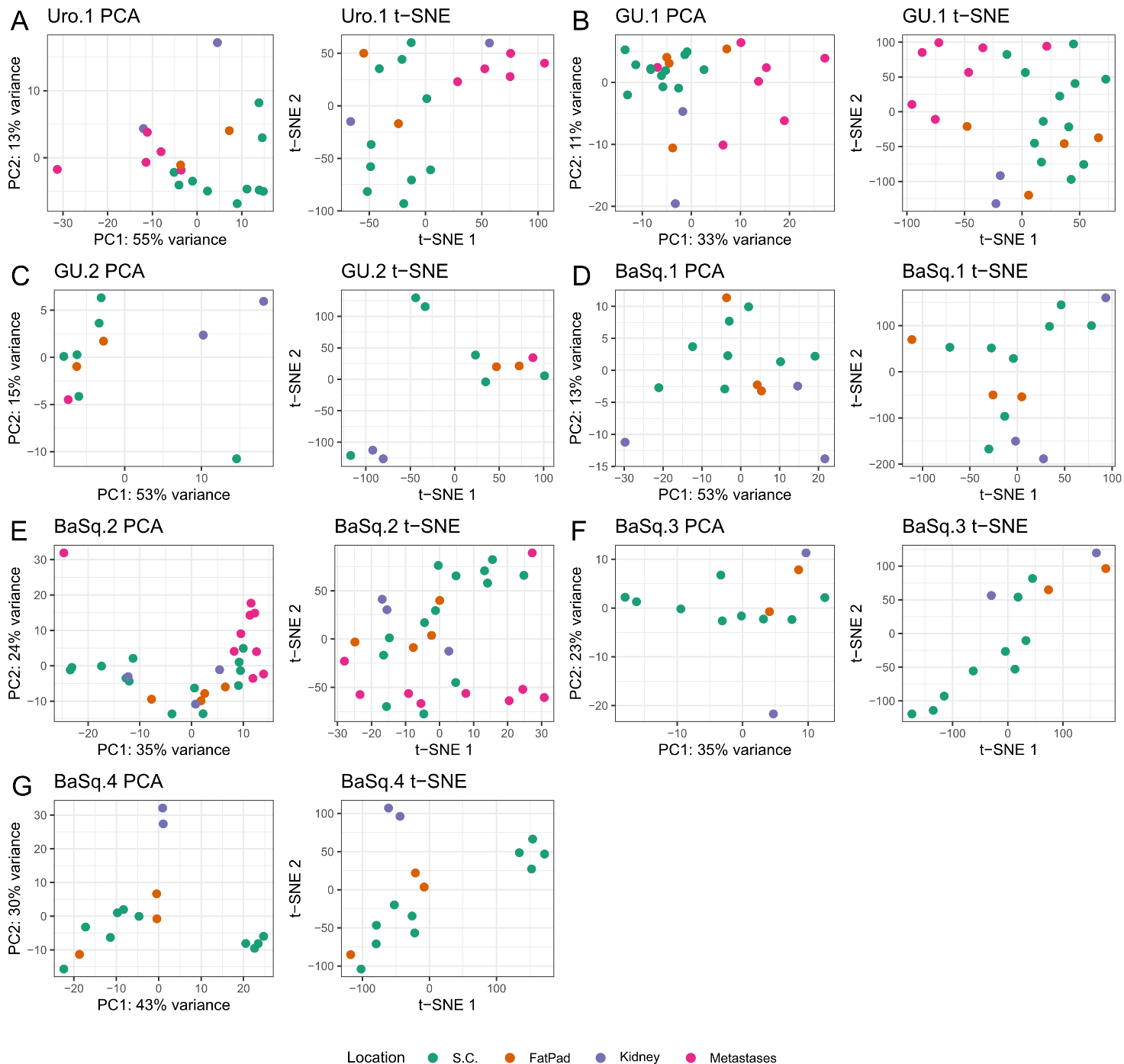


**Supplementary figure 3**. Clustering analysis of all samples within each model using principal component analysis (PCA), and t-distributed stochastic neighbor embedding (t-SNE) on the top 500 most varying genes within each model. Each dot represents a sample and is colored according to the growth location: subcutaneous (SC), fat pad (FP), under kidney capsule (KD) and metastatic locations (Mets).


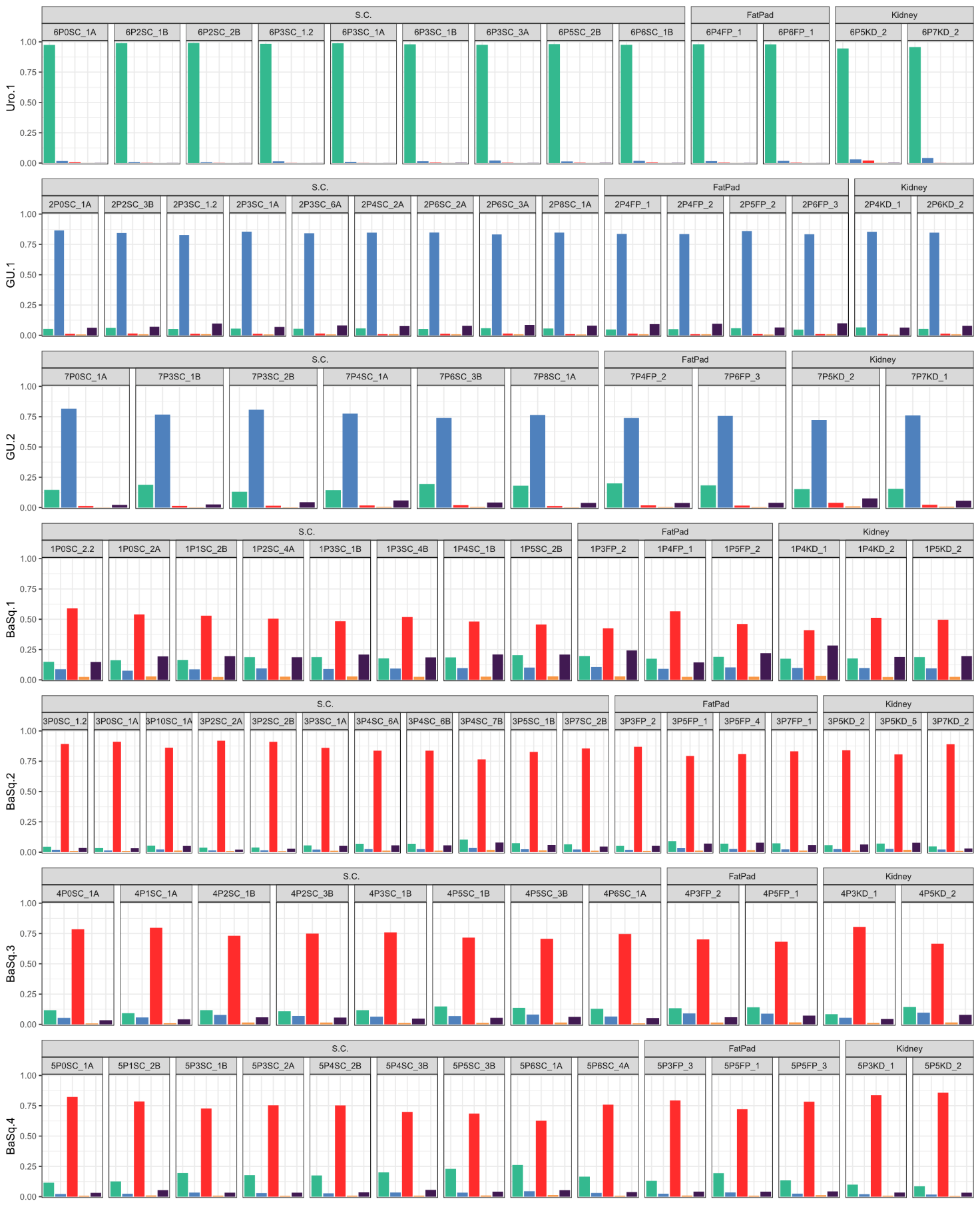


**Supplementary figure 4**. Barplots showing the molecular subtype scores for each sample across the different models and different tumor inoculation sites: S.C.: subcutaneous, Fat Pad and under Kidney capsule. Each panel presents a different model as indicated on the left side of the plots. The score profiles remain remarkably stable across the different microenvironments in most models, with minor fluctuations in some samples of the basal models.


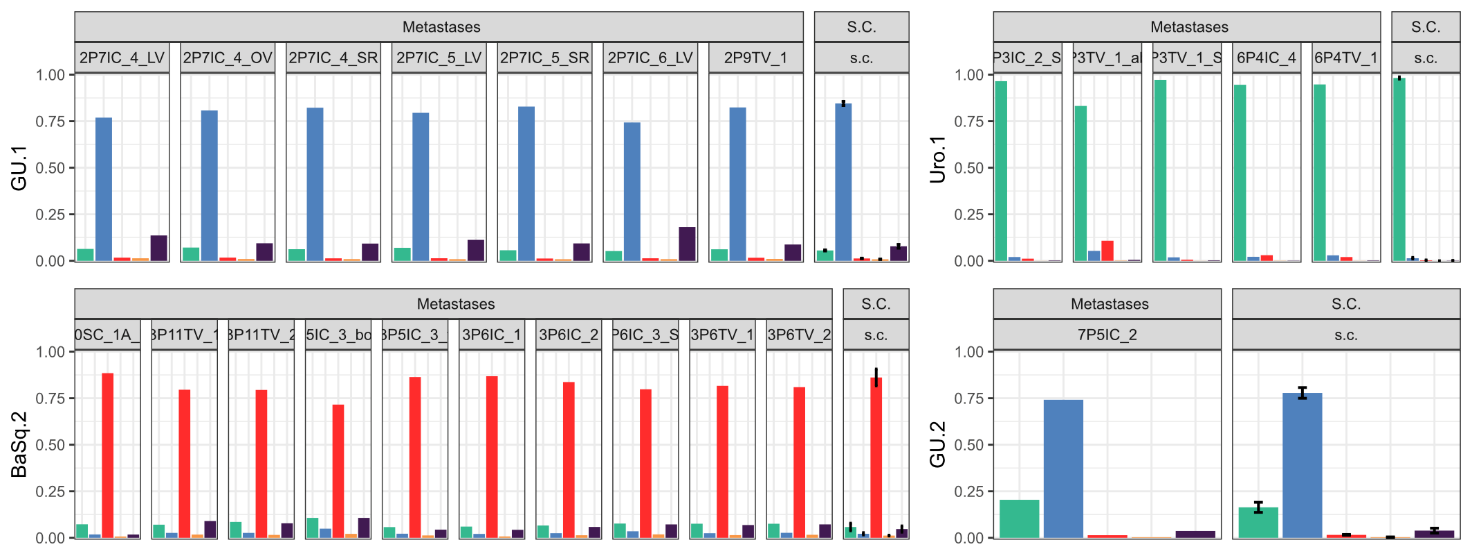


**Supplementary figure 5.** Barplots showing the molecular subtype scores for each metastasis sample compared with the average score of the corresponding S.C. samples. Each panel presents a different model, as indicated on the left side of the plots. The score profiles of the metastases samples are identical to the tumor growing in the subcutaneous space.


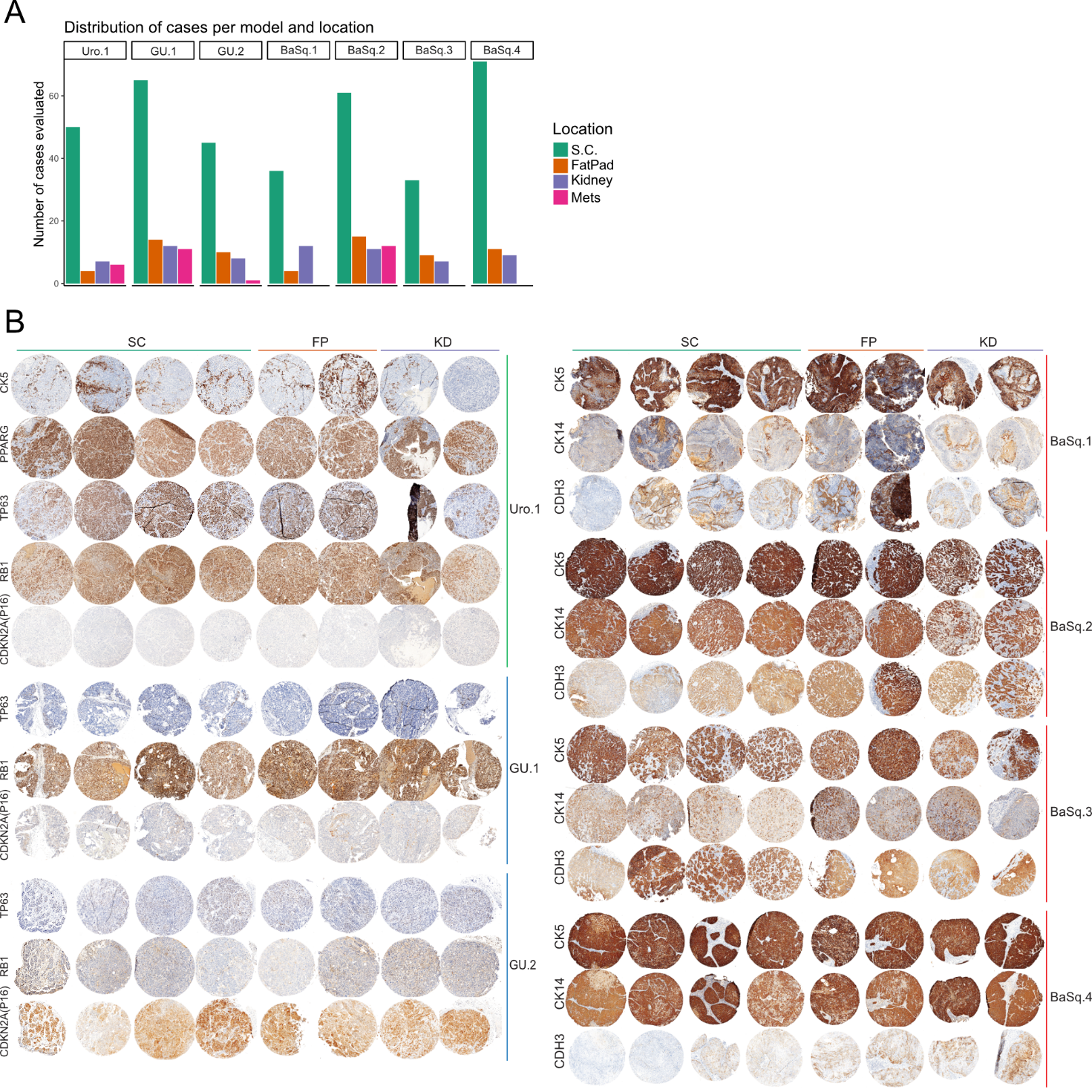
**Supplementary figure 6**. Bar plot showing the number of tumor samples evaluated by immunohistochemistry by model and location (A). Representative images of immunohistochemistry stainings across the different models showing the early, intermediate and last passage in subcutaneous space and the first and last passage in FatPad and Kidney as indicated by the top horizontal bars (B).


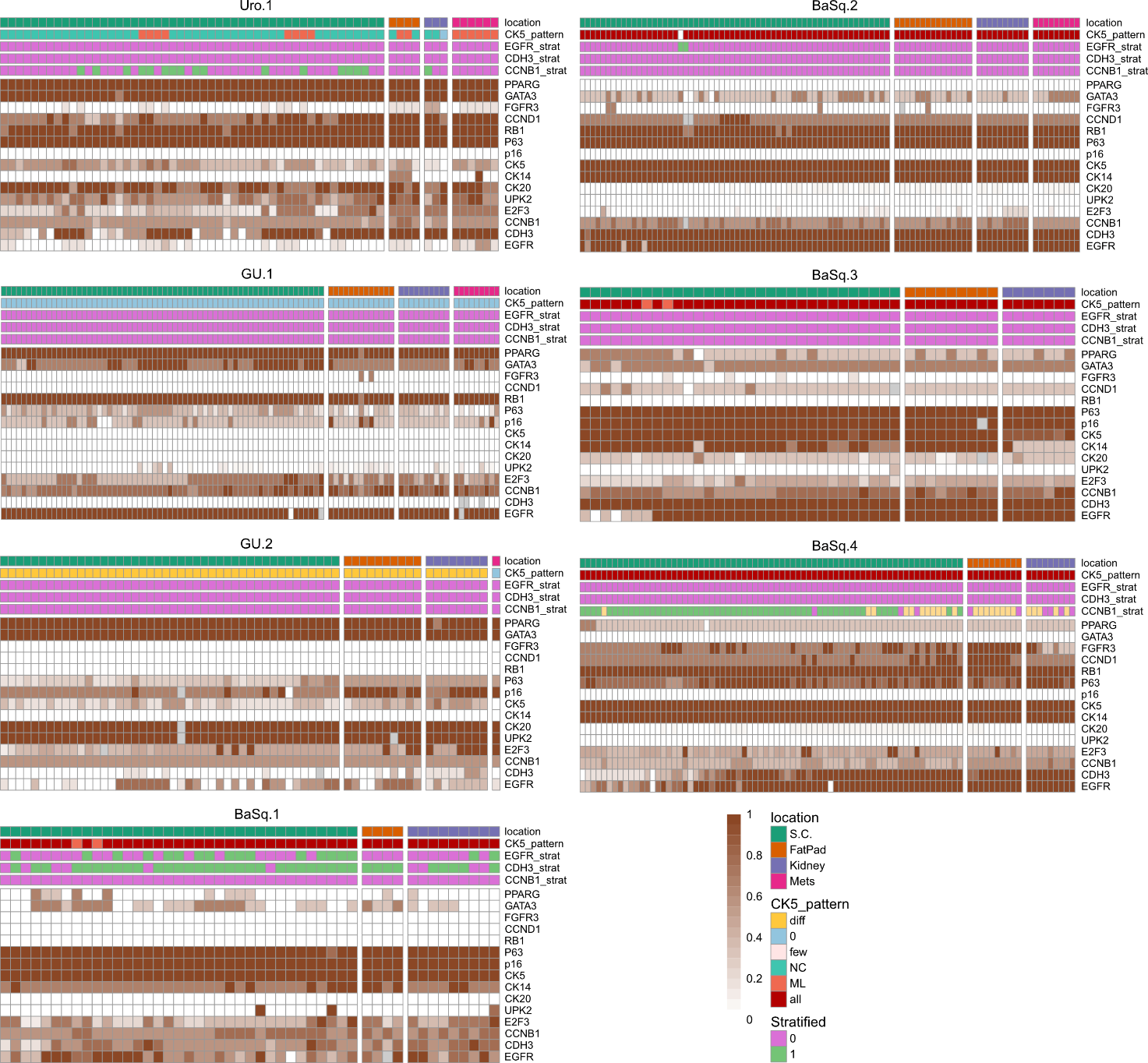


**Supplementary figure 7**. Heatmaps show the normalized IHC score for each marker for all models and locations; samples are sorted by model, molecular subtype, and location as shown in the top panel. The top panel shows the staining pattern for CK5 and stratification levels for EGFR, CDH3, and CCNB1, with 0 indicating diffuse and 1 stratified staining, i.e., close to the base membrane.


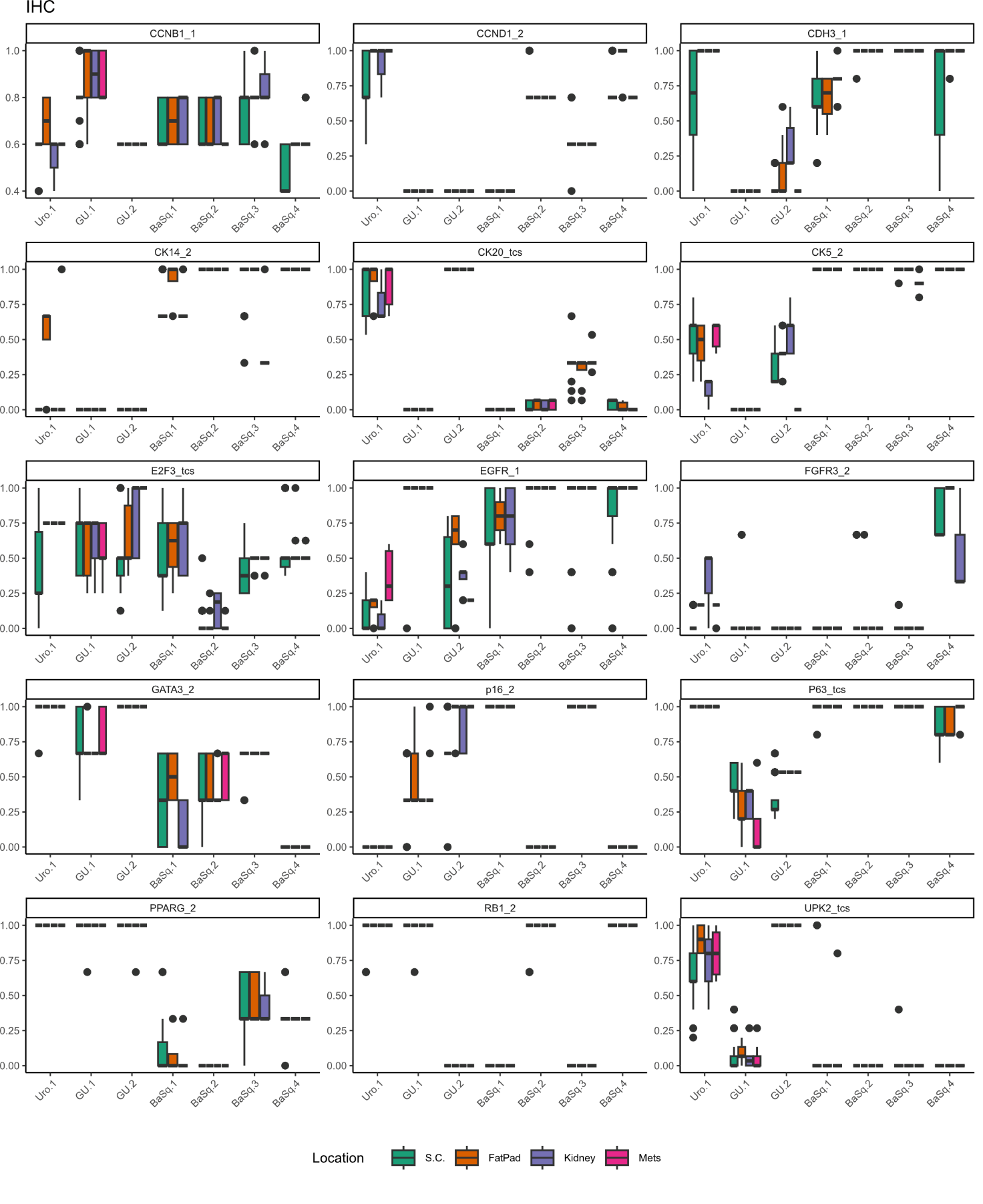


**Supplementary figure 8**. Boxplots showing the average expression level of the IHC markers for each model divided by location. List of markers: CK5, CK14, CK20, GATA3, PPARG, CCNB1, CCND1, E2F3, p16, RB1, CDH3, EGFR, FGFR3, P63, UPK2. All markers evaluated as proportion of positive cells (0-5) and intensity of staining (0-3). Tumor cell score (TCS) calculated as Intensity * Proportion/15. The title of each plot indicates the marker, and the suffix indicates the measurement with _1 for proportion, _2 for intensity, and _TCS for tumor cell score.


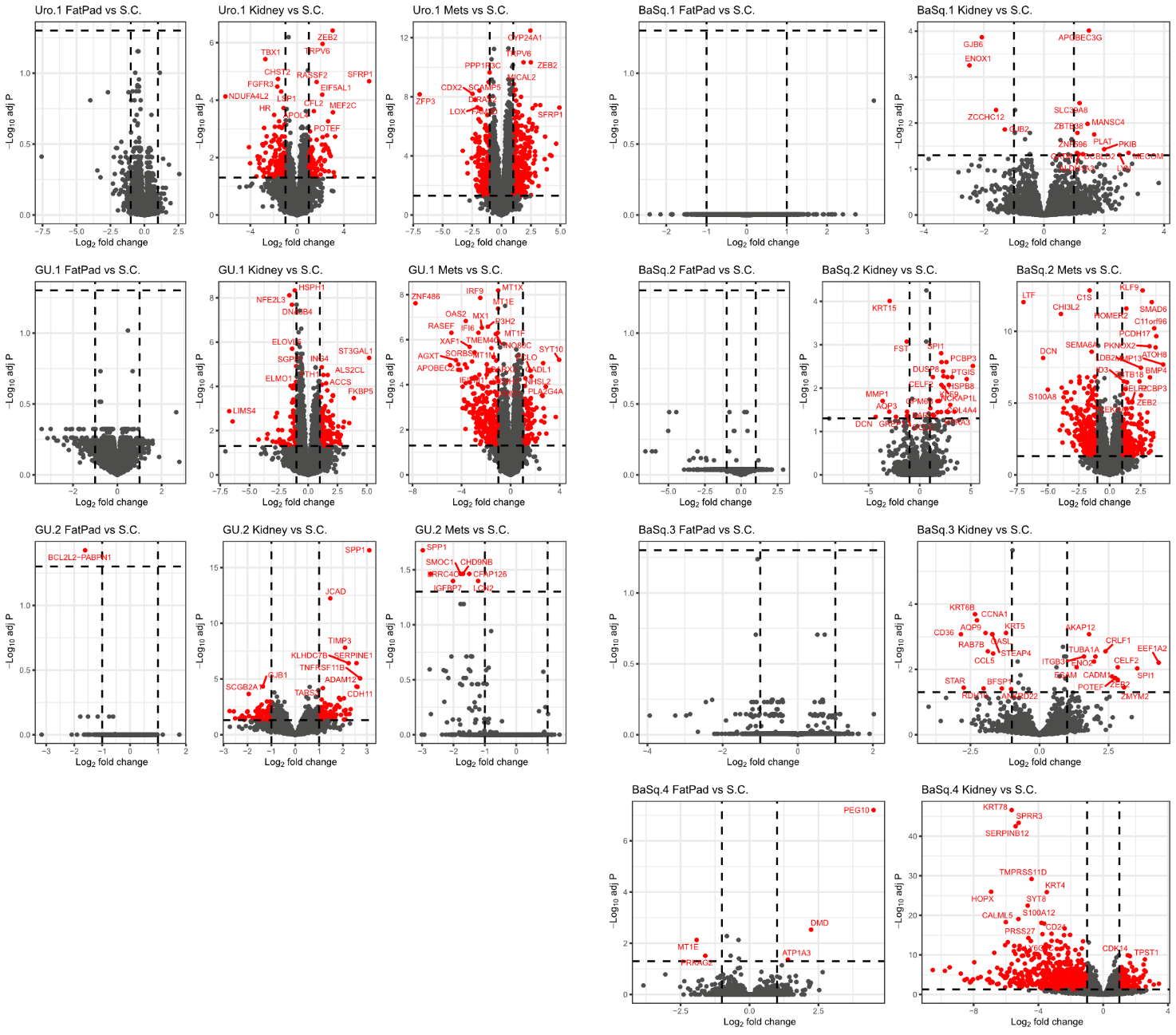


**Supplementary figure 9**. Differentially expressed genes (DEGs) after pairwise comparison between tumors from different locations and subcutaneous tumors across the different models. Genes with FoldChange>=1 and significant p-value are colored Red. The horizontal lines indicate false discovery rate (FDR) of 5% and vertical lines indicate log2 fold change -1 and 1.


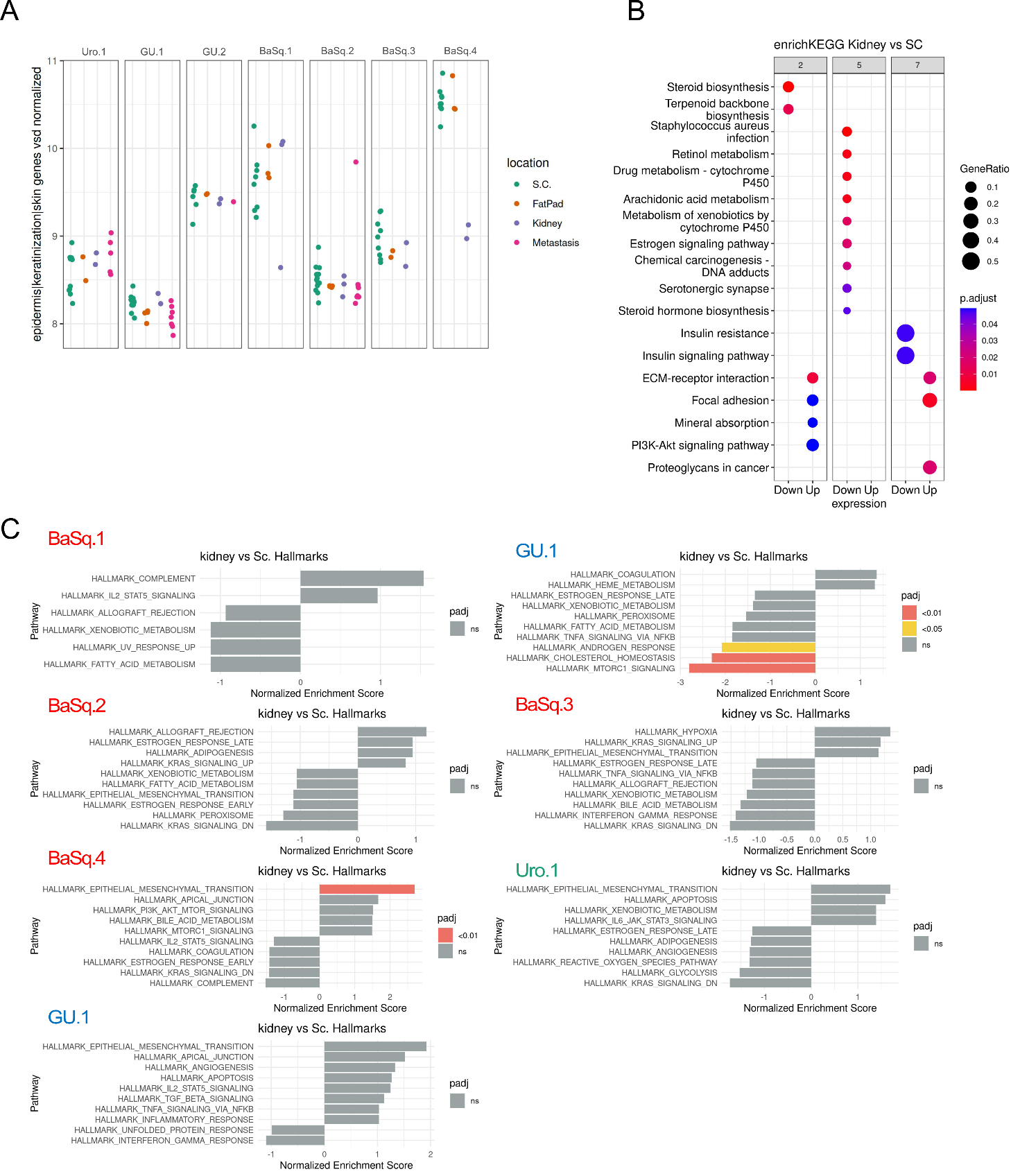


**Supplementary Figure 10**. Normalized expression levels of keratins-related genes across the models and locations (A). Enrichment analysis of DEGs between tumors grown under the kidney capsule vs subcutaneous. KEGG enrichment analysis of the gene sets showing shared processes enriched among the different models (B) and FGSEA enrichment of hallmarks pathways (C).


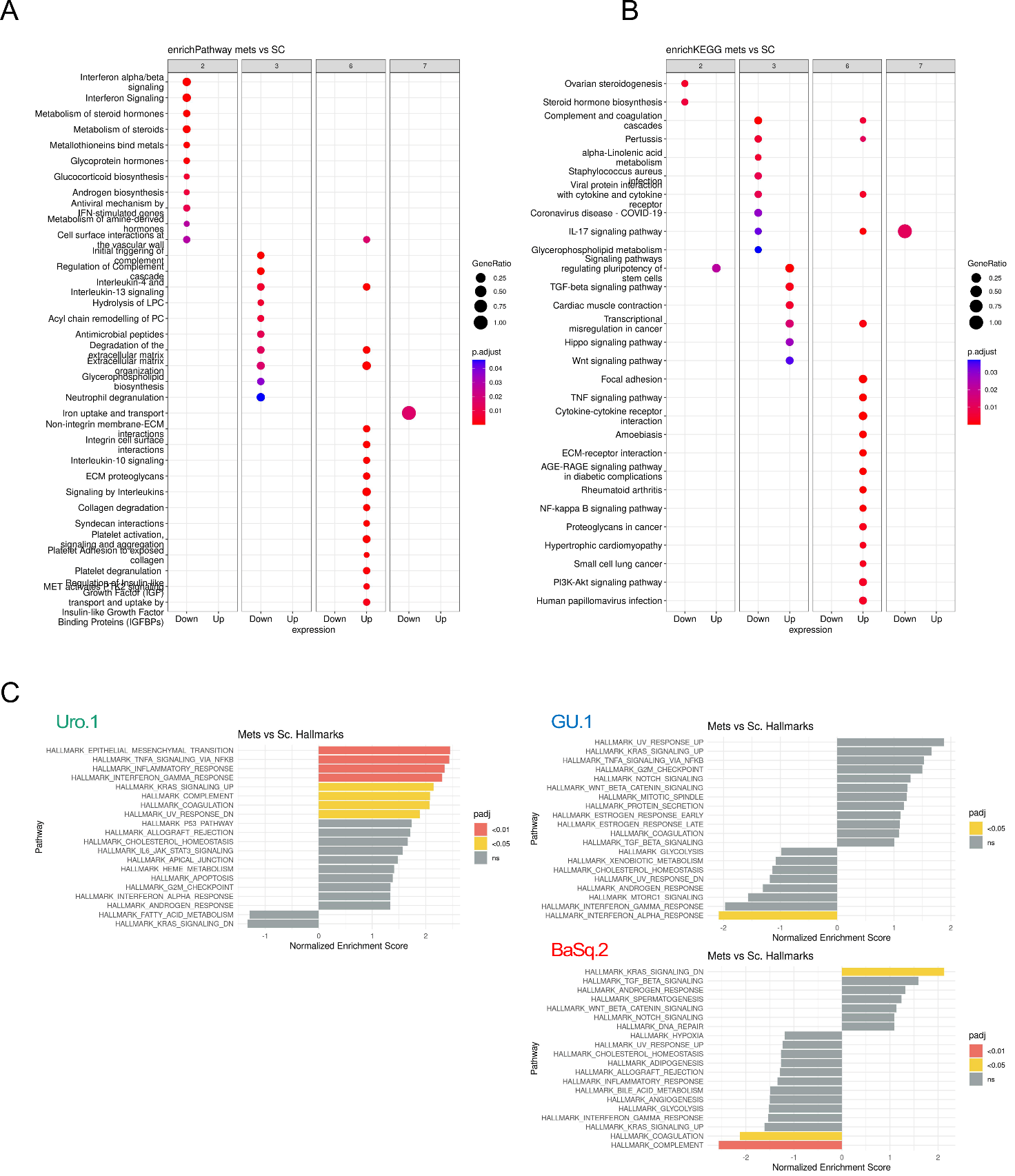


**Supplementary Figure 11**. Enrichment analysis of DEGs between metastases and subcutaneous tumors. Pathway enrichment analysis of the gene sets showing shared processes enriched among the different models (A). KEGG enrichment analysis of the gene sets showing shared processes enriched among the different models (B) and FGSEA enrichment of hallmarks pathways (C).


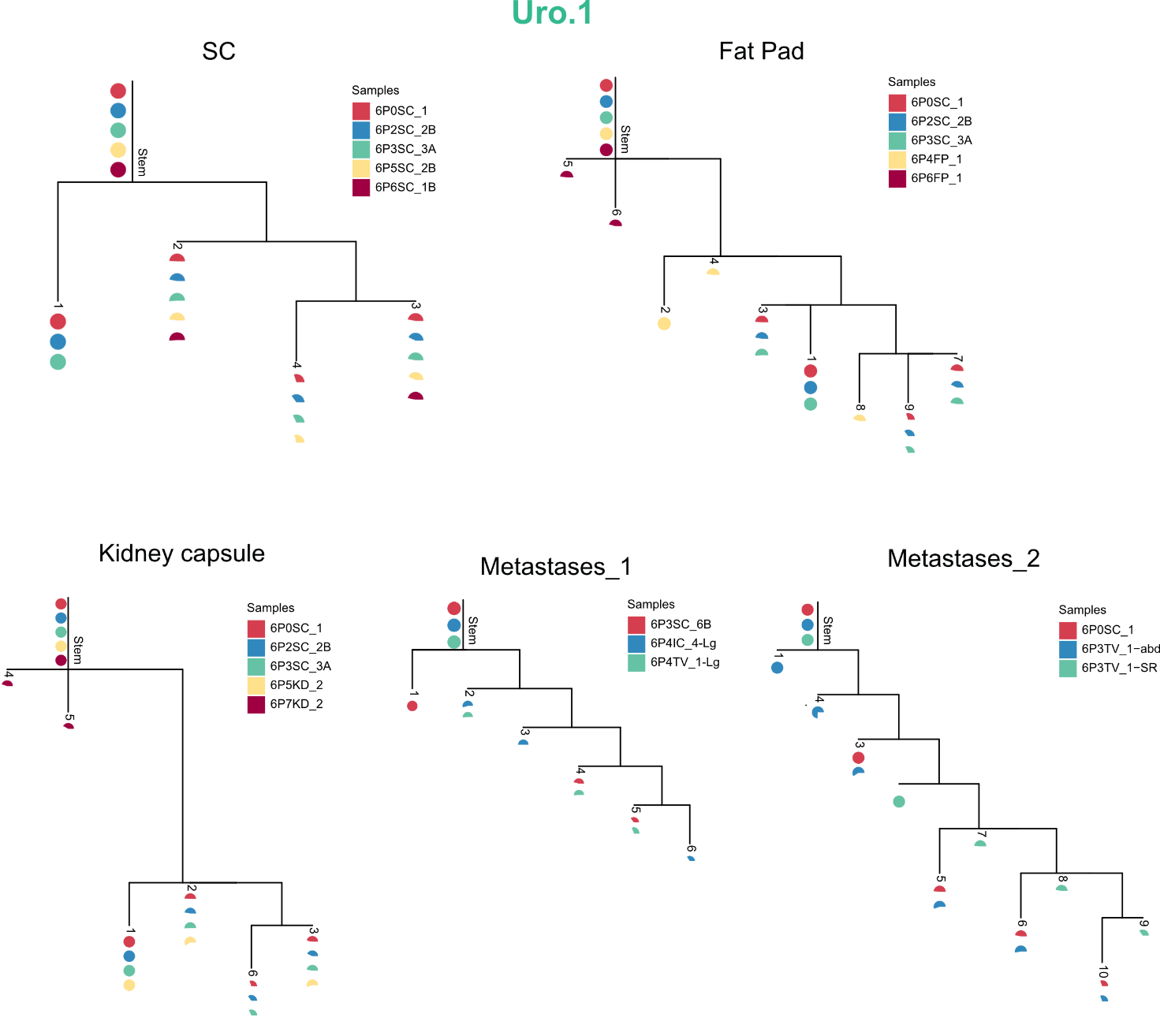
**Supplementary Figure 12.** Phylogenetic trees illustrating the clonal evolution within the Uro model across the different tumor sites. As indicated in the subtitles, each tree contains samples with a direct relationship and from a specific lineage (See supplementary figure 1). At the top of the tree is the stem, which represents the genetic alterations shared by all cells across all biopsies. Adjacent to the stem, the biopsies included in the tree are depicted as filled pie charts in different colors. The entities at the leaf nodes correspond to distinct groups of cells with unique genomic profiles (subclones), labeled with numbers. The branches indicate in which biopsies the subclones are found and their relative proportions within those biopsies.


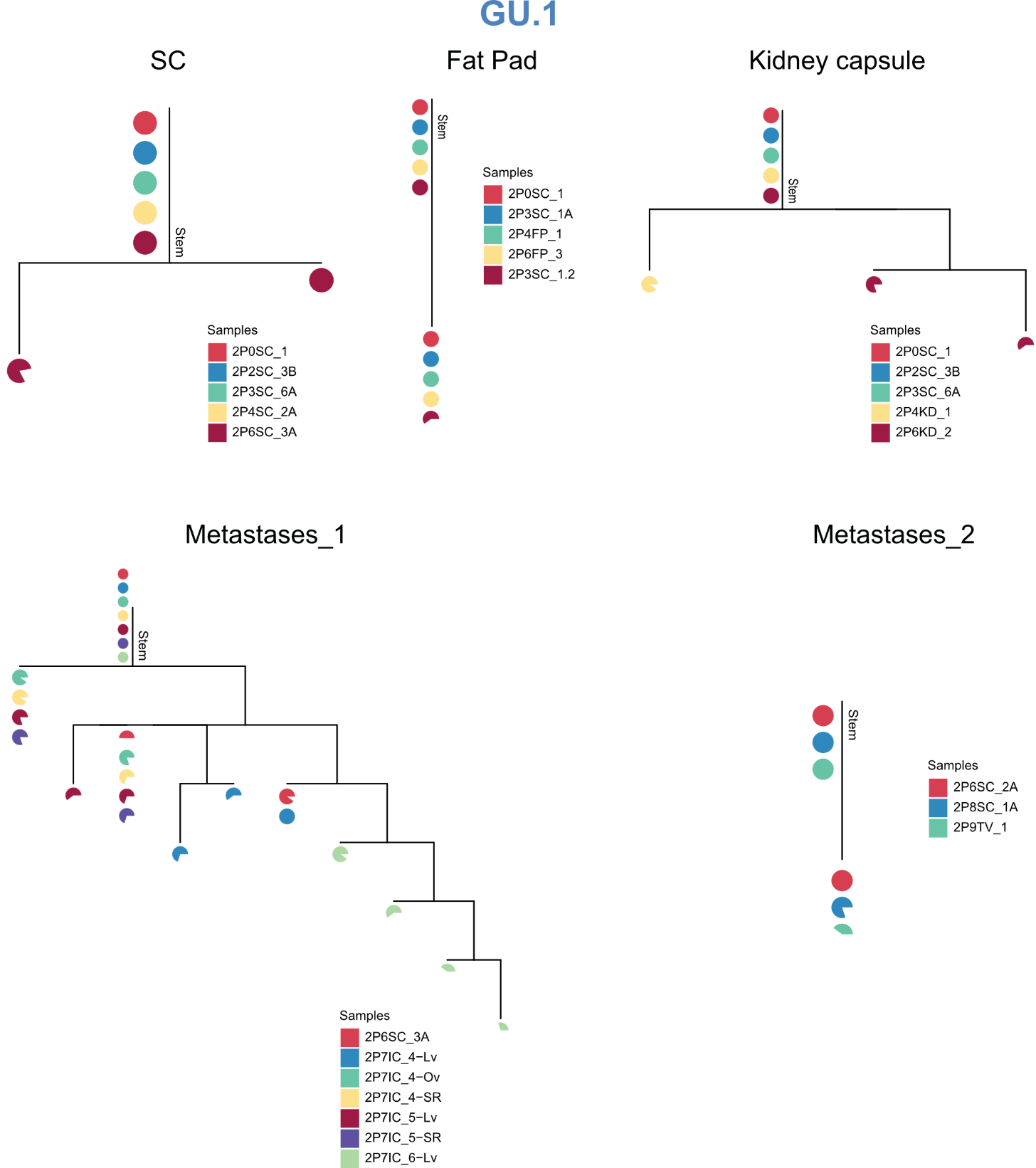


**Supplementary Figure 13.** Phylogenetic trees illustrating the clonal evolution within the GU.1 model across the different tumor sites. As indicated in the subtitles, each tree contains samples with a direct relationship and from a specific lineage (See supplementary figure 1). At the top of the tree is the stem, which represents the genetic alterations shared by all cells across all biopsies. Adjacent to the stem, the biopsies included in the tree are depicted as filled pie charts in different colors. The entities at the leaf nodes correspond to distinct groups of cells with unique genomic profiles (subclones). The branches indicate in which biopsies the subclones are found and their relative proportions within those biopsies.


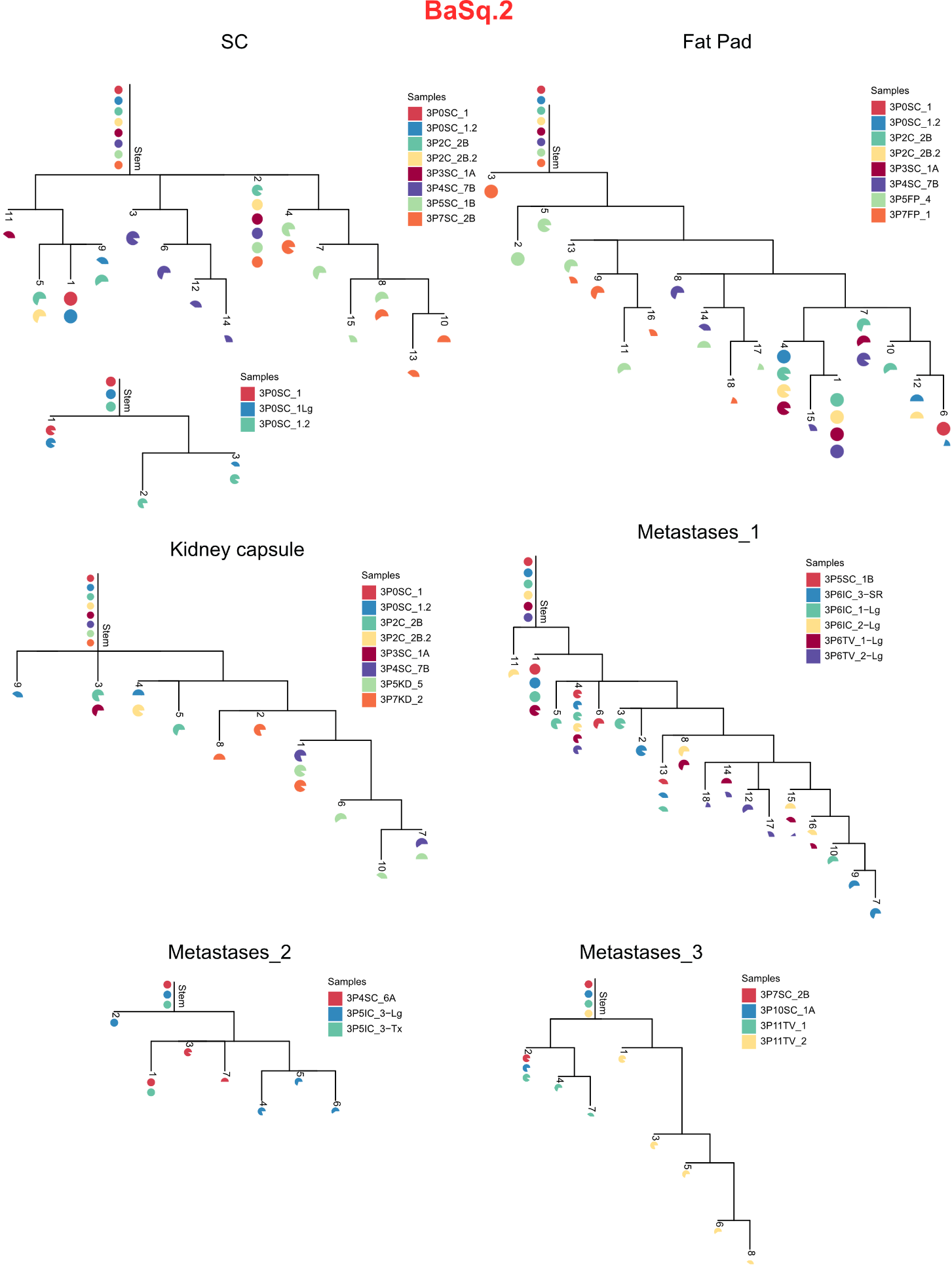
**Supplementary Figure 13.** Phylogenetic trees illustrating the clonal evolution within the BaSq.1 model across the different tumor sites. As indicated in the subtitles, each tree contains samples with a direct relationship and from a specific lineage (See supplementary figure 1). At the top of the tree is the stem, which represents the genetic alterations shared by all cells across all biopsies. Adjacent to the stem, the biopsies included in the tree are depicted as filled pie charts in different colors. The entities at the leaf nodes correspond to distinct groups of cells with unique genomic profiles (subclones), labeled with numbers. The branches indicate in which biopsies the subclones are found and their relative proportions within those biopsies.
